# Synthesis and Characterization of Catechol-Containing Polyacrylamides with Adhesive Properties

**DOI:** 10.3390/molecules27134027

**Published:** 2022-06-23

**Authors:** Kathleen Hennig, Wolfdietrich Meyer

**Affiliations:** Fraunhofer Institute for Applied Polymer Research IAP, 14476 Potsdam, Germany; Kathleen.hennig@iap.fraunhofer.de

**Keywords:** catechol, acrylamides, mussel-inspired adhesives, biomimetic

## Abstract

In this study, a row of four analogous dopamine acryl- and methacrylamide derivatives, namely *N*-(3,4-dihydroxyphenyethyl) acrylamide, *N*-(3,4-dihydroxyphenyethyl) meth acrylamide, *N*-phenethyl methacrylamide, *N*-(4-hydroxyphenethyl) methacrylamide were synthesized and characterized by ^1^H-NMR and ^13^C-NMR, followed by further solvent-based radical polymerization with *N*-hydroxyethyl acrylamide. All copolymers were characterized by ^1^H-NMR, dynamic differential calorimetry, and gel permeation chromatography. The dependency of the used comonomer ratios to the molecular mass of the corresponding copolymers has been described. The synthesis of the various polymers serves as a feasibility study and provides important data for a future biometric application in the medical field. We synthesized *N*-(3,4-dihydroxyphenyethyl) acrylamide copolymer up to 80 mol% by free radical polymerization without using any protecting groups. All polymers show identical perfect adhesive properties by a simple scratch test. Further, the monomers were used as a photo reactive glue formulation to test its adherence to a medical titanium surface sample by tensile shear test.

## 1. Introduction

Mussels have the unique ability to adhere to a variety of polar and non-polar surfaces such as glass or fluorinated polymers (Teflon) in aqueous environments [1,2]. The unique adhesion property originates from the catechol function of the amino acid 3,4-dihydroxyphenylalanine (DOPA) [2,3]. In recent years, many catechol-based polymeric materials have been developed for surface modification applications and the development of coatings with numerous applications. Catechols and their derived compounds can self-assemble on various inorganic and organic materials, including metals, metal oxides, mica, silica, ceramics, and polymers [4]. Catechol polymers are of particular interest for biomedical applications due to their high adhesiveness in aqueous environments [5]. Biomedical applications proposed for catechol polymers include structural adhesives for tissue [6] and bone [7,8,9], denture adhesives [10,11], self-healing and stimulant materials [12,13,14], antibacterial [15] and antifouling materials [16,17].

Mussel adhesive proteins (MAPs) provide an innovative approach to develop new polymers for biomedical applications in the body. One strategy to prepare synthetic polymers with catechol moieties on the side chains is free radical polymerization [12,14,17,18,19]. Recent smart coating materials mostly contain acrylamides as co-monomers, such as poly(*N*-isopropylacrylamide), to create functionalized surfaces, e.g., with structured nanopillar array properties [20] and thermoresponsive hydrophobic interaction [21] or stimulatory properties [22] such as cell attachment/detachment [23].

Here, we synthesized catechol-containing statistical copolymers for biomimetic coating material A variety of differently composed copolymers of *N*-(3,4-dihydroxyphenethyl) meth acrylamide (DMA) and *N*-(2-hydroxyethyl) acrylamide (HEAA) were synthesized by copolymerization. HEAA was used as a comonomer based on its good resistance to bacterial adhesion and protein adsorption with long-term biocompatibility [24,25]. A combination of HEAA with other comonomers as mixed polymer brushes also exhibit strong antifouling properties [26]. We made use of this property to synthesize new adhesive antifouling materials with catechol-containing acrylamides. (*N*-(3,4-dihydroxyphenyethyl) acrylamide (DA) and DMA were used as monomers to investigate the impact of the methyl group on the vinyl function during polymerization. In addition, monomers without or with only one hydroxyl group on the phenyl ring were synthesized. Here, we investigated the effect of the hydroxyl groups on the phenyl ring regarding the accessibility of the monomers and their yield as well as their polymerization. Finally, the polymers were synthesized for a later biometric application in the medical field. The synthesis of the various polymers was used as the first step for a feasibility study. Such studies are used to gain initial information about the synthesis of the polymers and to investigate the influence of the comonomer content and molecular weight on the material properties [21]. The monomer HEAA is readily provided with hydroxyl groups in the side chain. The aim of the study was to show the impact of the hydroxyl groups at the side chain end of the synthesized monomers on the yield of the HEAA copolymers. Can the monomers form copolymers with the HEAA monomer and up to which maximum content is this possible? Is it also possible to synthesize homopolymers? With this study, we were able to answer all of our questions and determine the importance of the hydroxyl and methyl groups in polymerization and a first indication of the adhesion efficiency.

## 2. Results and Discussion

### 2.1. Synthesis and Characterization of Monomers

**Figure 1:**
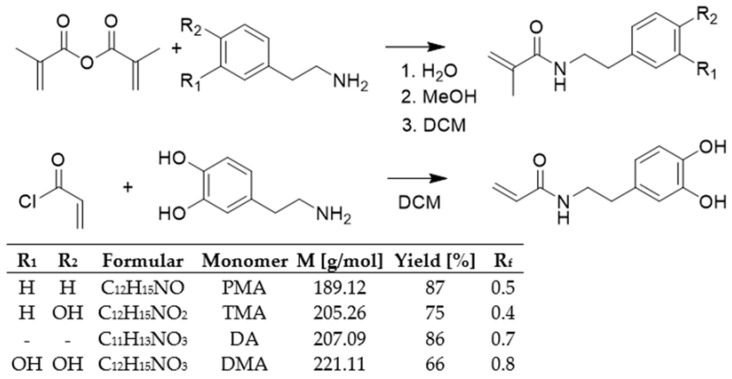
Synthesis of the monomers DMA (1), TMA (2) PMA (3) and DA. Molecular formula, molar masses, and yields.

All monomers were accessible and could be synthesized as shown in Figure 1. The yield of the methyl monomers decreased with an increase in the hydroxyl groups on the phenyl ring. The synthesis of DMA has been described in the literature [8]. Monomers were synthesized as follows: DMA (66% in yield) was obtained by reaction of dopamine hydrochloride with methacrylate anhydride. TMA (75% in yield) was obtained by reaction of tyramine with methacrylate anhydride in methanol. PMA in DCM at a yield of 87% was obtained by reaction of phenylethylamine and methacrylate anhydride. A similar high yield was also obtained for the DOPA monomer without a methyl group on the vinyl group. The reaction of dopamine hydrochloride with acryloyl chloride produced DA with 86% in yield. The reaction was carried out in a borate buffer to protect dihydroxy benzene moiety.

### 2.2. Synthesis and Characterization of Polymers

**Figure 2:**
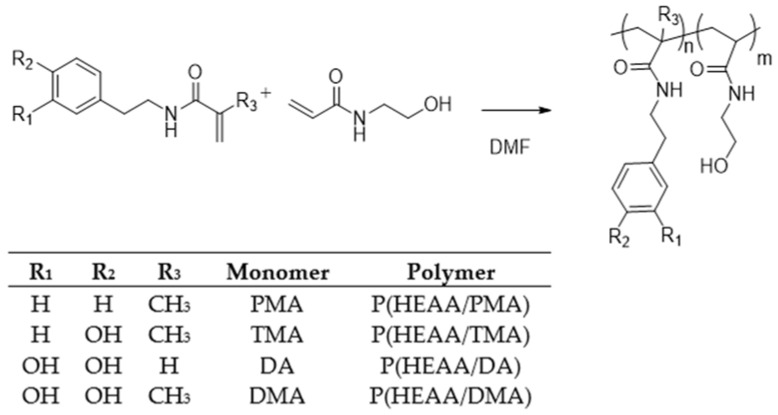
AIBN initiated polymerization with dopamine-modified acrylamides and HEAA to copolymers.

Poly(HEAA/DMA) were synthesized by the free-radical polymerization method, using a different molar ratio of HEAA/monomer and 1% azobisisobutyronitrile (AIBN). P(HEAA) homopolymer was also synthesized as a control polymer under identical conditions. In order to investigate the impact of the catechol function on adhesion, comparative polymers P(HEAA/PMA) and P(HEAA/TMA) were prepared as is shown in Figure 2 in summary.

#### 2.2.1. H-NMR Analysis

**Figure 3:**
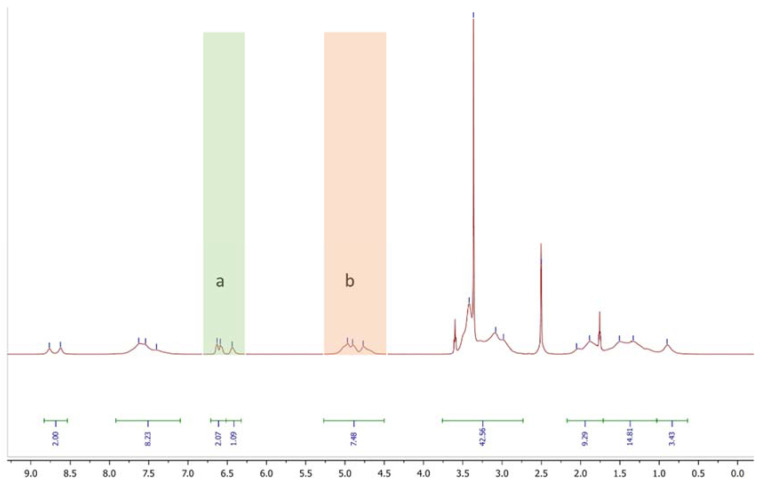
^1^H-NMR (DMSO-d_6_, 500 MHz, 298 K) of P(HEAA/DMA_10_), (**a**) peaks of aromatic DMA protons, (**b**) peaks of OH HEAA protons.

Figure 3 shows the ^1^H-NMR spectra of P(HEAA/DMA_10_) polymer and the integral areas of various resonance peaks of protons. The double peak at 8.70 ppm can be attributed to the OH-protons of DMA. The peaks at 7.54 ppm are the result of the amide protons of both monomer parts. The peaks at 6.44–6.63 ppm (a) can be attributed to the aromatic protons of DMA. The peaks at 4.90 ppm (b) are caused by the OH proton of HEAA. The ratio of the integral area at peak (a) and peak (b) with 12 mol% of DMA is near the designed one of 10 mol% (see Supplementary Materials). The compositions of all synthesized polymers are close to the designed composition (Table 1). The difference of feed ratio to polymer is highest for the DMA. 2–3% more DMA was integrated compared to the feed ratio. For the PMA monomer, it is balanced. There was 1% more incorporation for P(HEAA/PMA_30_) and 1% less for P(HEAA/PMA_10_). For TMA and DA monomers, the feed ratio is equal to the polymer composition or 1–2% less in the polymer. The spectra of all components are presented in the Supplementary Materials.

#### 2.2.2. Molecular Weight

The results of the syntheses are shown in Table 1. Polymers with relative yields in the wider range of ~0–90% were obtained. The number average molecular weights M_n_ of all synthesized copolymers ranges from 3.8–12.4 kg mol^−1^ with polydispersity index PDI of 1.7–2.7. Except for the polymer P(HEAA/DMA_10_) with higher M_n_ 30.1 kg mol^−1^. The other two copolymers with 20 mol% and 30 mol% DMA share the same M_n_ of 3.8 kg mol^−1^, corresponding to 28 and 23 monomer units. In comparison, Payra polymerized DMA by free-radical polymerization with acrylates with similar PDIs between 1.7–2.7. The molecular weight distributions of the polymers (M_W_) ranged from 20 kg/mol to 111 kg/mol [19]. Our polymers showed a much lower M_w_. Payra obtained the highest M_W_ of 111 kJ/mol with the lowest DMA content of 1:100 [19]. We could confirm this in our experiment as well, the highest M_W_ was obtained with the lowest DMA content of 10 mol%. Yabu et al. polymerized DMA with styrene or *N*-dodecylacrylamide and also achieved a low M_n_(PDI) of 7.9(2.23) and 6.8(1.68) similar to ours [27]. Polymers with higher DMA content could not be synthesized. This is consistent with the literature. Homopolymers of dopamine could previously only be synthesized by protecting the catechol function during polymerization and subsequent deprotection [15,28]. Free radical polymerization of polymers without protection of catechol function are known up to a content 48 mol% [29]. The homopolymer p(HEAA) has a similar molecular weight of M_n_ = 2.5 kg mol^−1^. Thus, the low molar masses of the polymers are not due to the dopamine-modified monomer but to the low reactivity of the acrylamide monomers. The polymerizations of acrylamide in non-aqueous solvents proceed only at low rates, resulting in polymers with low molar masses [30,31]. Non-aqueous solvents stabilize the radical electron by conjugation over the amide bond, leading to a decrease in reactivity. Reactivity is also reduced by association of the monomers to form di-, trimers and linear multimolecular structures [30,31].

No homopolymers P(DMA/TMA/PMA/DA) could be synthesized. The dopamine acrylamide DA showed the highest reactivity under the same experimental conditions. A P(HEAA/DA) copolymer could be isolated up to an acrylamide content of 80 mol%. This corresponds to a catechol content of 88 wt%. We synthesized DA copolymer up to 80 mol% by free radical polymerization without using any protecting groups. The polymer properties will differ only slightly from those of a pure homopolymer. This saves the step of protecting and deprotecting during synthesis.

**Figure 4:**
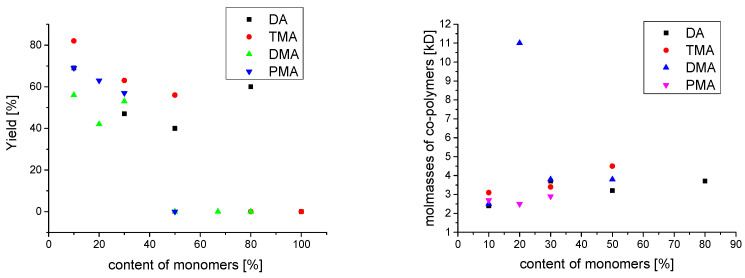
Dependencies of the yield of the corresponding copolymers (**left**) and M_n_ (average molecular weights) of the copolymers on the monomer composition in mol percentage (**right**).

With a monomer composition of 1:1, both polymer with DA and TMA could be copolymer synthesized. The dopamine and phenyl monomer formed an isolatable copolymer only at a higher HEAA content of 70 mol%. It is surprising that here, too, no polymers can be isolated at a PMA content of 50 mol%. The OH groups of the phenyl side chain of TMA and DMA were expected to have an inhibitory effect on free-radical polymerization. Phenolic compounds such as catechol are known for their inhibitory effect [32]. Due to the absence of the OH group in PMA, a higher reactivity of the monomer was supposed. This was not confirmed. Steric hindrance or influence of the hydroxyl groups at the phenyl ring on polymerization is not obvious. In general, a decrease in yield and an increase in M_n_ of the copolymers are observed as a function of monomer concentration (Figure 4). The polymers of PMA without hydroxyl group showed the lowest PDIs and M_n_s compared to the other methacrylamide polymers. The polymers p(HEAA/PMA) have a low number average molecular weights of 2.5–2.9 kg mol^−1^ and PDI of 1.8–2.0. The yields decrease and M_n_ of the polymers increase with increasing PMA content. The degree of polymerization remains stable at around 20 monomers according to the higher PMA monomer weight.

The number-average molecular weights M_n_ of the polymers (P(HEAA/TMA)) increase with higher content of TMA from 3.1 to 4.5 kg mol^−1^. The number of monomer units changes only slightly from 25 to 28. The yields of the copolymers decrease with rising TMA content. The polydispersity index (PDI) of the polymers is in the range of 2.1–2.7.

The dopamine acrylamide DA showed a slightly higher reactivity under the same experimental conditions because of the isolable P(HEAA/DA_80_). The number average molecular weights (M_n_) of the P(HEAA/DA) copolymers increases on average by increasing the DA content from 2.4 kg mol^−1^ to 3.7 kg mol^−1^. The degree of polymerization is approximately 20 units for all copolymers. The polydispersity index ranges from 1.7 to 2.1 and is comparable to the values of the PMA polymers. PDI values and M_w_ were lower in direct comparison to the corresponding DMA with the methyl group.

The glass transition temperature (T_g_) is highest for the homopolymer P(HEAA) at 176.0 °C. It is much lower between 99.9 °C to 131.1 °C for all copolymers. The glass transition temperatures of the polymers increased with the number of phenolic hydroxy group. The lowest value was determined for P(HEAA/PMA_20_) with 99.9 °C, T_g_ is 110.5 °C for P(HEAA/PMA_10_) as well as P(HEAA/PMA_30_). T_g_ increases with increasing contents of DA and TMA in the copolymers from 123.4 °C for P(HEAA/DA_30_) to 128.9 °C for P(HEAA/DA_80_) as well as 111.3 °C for P(HEAA/TMA_10_) to 128.2 °C for P(HEAA/TMA_50_). In contrast, T_g_ decreases slightly with increasing content of DMA from 131.1 °C P(HEAA/DMA_10_) to 126.0 °C P(HEAA/DMA_30_). Payra’s acrylate copolymers exhibited higher T_g_ with increasing DMA content in contrast to our observations [19]. We could only confirm this behavior in our experiment with our DA and TMA copolymers.

The adhesion strength of the polymer coatings was measured according to DIN EN ISO 2409. All synthesized polymers adhere very well to the glass. All polymer film showed completely smooth cut edges and none of the squares of the grid were detached. According to these results, the cross-cut class 0 is to be assigned accordingly for all polymer coatings (Table 1).

#### 2.2.3. Photo-DSC of Monomers Formulation

**Figure 5:**
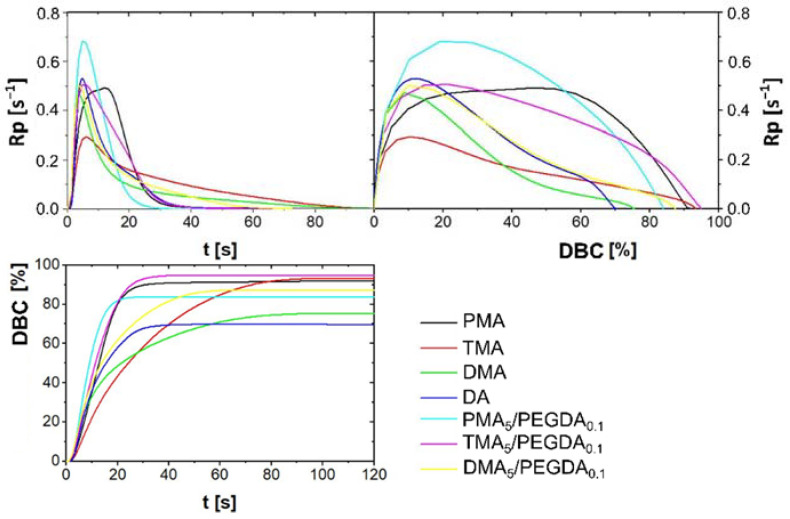
Photo-DSC of dopamine-modified acrylamides and HEAA/PEGDA to copolymers.

The photo-reactivity of the monomers has been analyzed by photo-DSC in isothermal mode. Each monomer was mixed with 95 mol% of HEAA in order to receive liquid adhesive that can be cured by UV light. 5 mol% of acrylamide and 0.5 wt% of the photoinitiator (PI) Irgacure 369 before analysis at 25 °C with a UV intensity of 1 W cm^−2^. The photo-curing rate (Rp) and double bond conversion (DBC) values as a function of time were calculated from photo-DSC data (presented in Figure 5 and Table 2).

The polymerization conversion was lowest for the dopamine with two hydroxyl groups with 75.4% for DMA. The conversion for TMA was 93.2% and for the phenyl monomer without hydroxyl group was almost identical with 93.4%. Initial rates were nearly identical for all monomers. The maximum polymerization rate (Rp_max_) for the TMA with 0.29 s^−1^ and Rp_max_ for PMA and DMA were very close to each other with values of 0.49 s^−1^ and 0.47 s^−1^. DA has shown the highest rate of 0.53 s^−1^ among the unbranched polymers. This is due to the higher reactivity of the formed radicals formed. The tertiary methyl acrylamide radicals are more stable and of lower reactivity than the secondary acrylamide radicals. The polymerization is completed after a conversion of 70%. The rapid retardation and low conversion rate of DA and DMA may be due to the inhibitory effect of the catechol group [33,34]. The polymerization rate decreased also in the case of TMA. However, the rate decreases only slowly. The polymerization rate of 5%-PMA-formulation is constant for about 20 s or a conversion of 60% and then decreases. The conversion rate was improved by using a PEGDA as an additional crosslinker, confirmed by photo DSC. We have decreased the content of HEAA to 94.9 mol% and added 0.1 mol% poly(ethylene glycol) diacrylate (PEGDA, M_n_ 10 kg mol^−1^). PEGDA as the additional crosslinker exhibited higher Rp_max_, t_max_, and higher conversions with the exception of PMA_5_/PEGDA_0.1_, which achieved a conversion of only 83.8%. The DBC could be increased to 87.3% by adding PEGDA to DMA, as well as the Rp_max_ to 0.50 s^−1^.

#### 2.2.4. Tensile Shear Test

**Figure 6:**
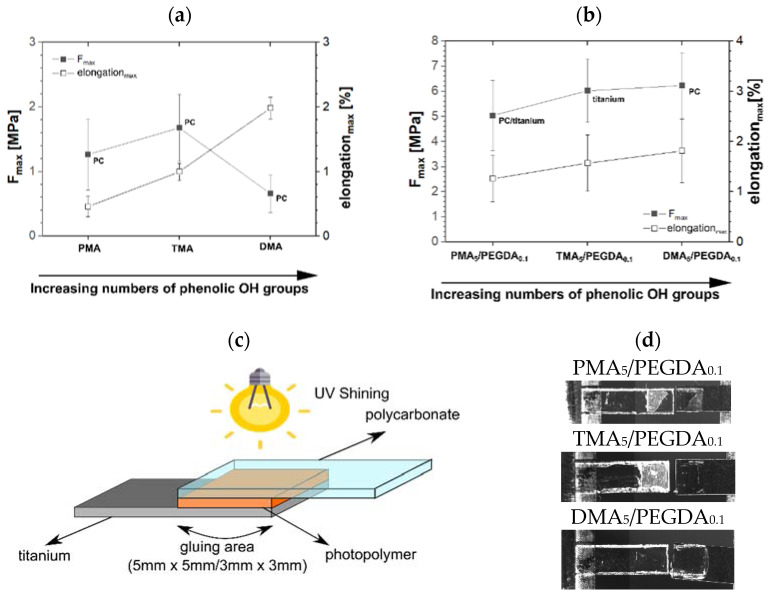
Tensile shear tests of photopolymers XMA (**a**) all adhesion fractures occurred to the PC surface) and photopolymers with crosslinker XMA_5_/PEGDA_0.1_ (**b**), Sample set up of tensile shear test (**c**) and destroyed samples (**d**).

The adhesion force of the glue formulations was measured by tensile shear tests. The glue compositions with described monomers were UV cured between light transparent polycarbonate (PC) titanium samples (Figure 6c). Adhesion fracture occurred on the side of the PC surface for all glue composition without any additional crosslinker as shown in Figure 6a. The photopolymer TMA showed the highest adhesive force with 1.68 MPa and a maximum elongation of 1.00%. The photopolymer PMA adhesion force to the PC surface was at 1.26 MPa and had a maximum elongation of 0.46% at break. The polymer DMA has shown the lowest adhesion force to the PC surface at 0.66 MPa and the highest elongation at 1.98%. The lower adhesion strength of DMA compared to the glue formulation with PMA and TMA is assumingly due to the lower DBC.

Better comparability of the samples was achieved by using additional content of PEGDA as the crosslinker in the formulation. The conversions have equalized and are now only between 87.3% and 94.8%. The samples are more comparable and have similar curing conversion to show the effects of the individual monomers. The gluing area had to be reduced from 15 to 9 mm^2^ due to the strong adhesion force to both surfaces. The maximum elongation of the materials was also more comparable and ranged from 1.27% to 1.81%. The adhesion fractures occurred for both surfaces for the polymers with PEGDA (Figure 6b). In contrast to the previous measurements, the adhesion fracture to the titanium interface occurred here for the polymer TMA_5_/PEGDA_0.1_ (Figure 6d). The measured force thus corresponds to the adhesion force to titanium and amounts to 6.02 MPa. The polymer PMA_5_/PEGDA_0.1_ showed a mixed type of cohesion and adherent breaks to both substrates, but most of the polymer remained on the PC surface. The maximum force reached 5.03 MPa and an elongation at break of 1.27%. Samples with the polymer DMA_5_/PEGDA_0.1_ partially showed adhesion fractures to both substrate sides, but most of the fractures occurred for the PC surface. The measured force of 6.23 MPa can therefore be interpreted as an adhesion force to the PC surface and its adhesion force to titanium is higher than this value. The maximum elongation was 1.81%. An improved adhesion to PC and titanium could be achieved by DMA with PEGDA. The adhesion was enhanced by catechol function compared to the other monomers with a hydroxyl group or without. The maximum elongation increased with the hydroxyl group number of the monomers for both polymers with and without PEGDA.

## 3. Materials and Methods

### 3.1. Materials

2-Phenethylamine (Sigma-Aldrich, St. Louis, MO, USA, 99%), tyramine (Sigma-Aldrich, 98%), dopamine hydrochloride (Sigma-Aldrich, 98%), *N*-hydroxyethyl acrylamide (Sigma-Aldrich, 97%), 2,2′-azobis(2-methylpropionitrile) (AIBN, Sigma-Aldrich, 98%), acryloyl chloride (Sigma-Aldrich, 97%), methacrylic anhydride (Sigma-Aldrich, 94%), polyethylene glycol diacrylate (PEGDA, Sigma-Aldrich, M_n_ 10 kg mol^−1^, 97%) and sodium borate decahydrate (Sigma-Aldrich, 99.5%) were used without further treatment.

### 3.2. Characterization

#### 3.2.1. Chromatography

The reactions were monitored by TLC (Thin-layer chromatography) performed by using POLYGRAM^®^ SIL G/UV254 from Macherey-Nagel, Düren, Germany. Column chromatography was performed by using Merck silica gel (particle size: 0.040–0.063 mm), Darmstadt, Germany.

#### 3.2.2. Gel Permeation Chromatography (GPC)

Average molecular weights and molecular weight distributions (polydispersity index PDI) of the produced polymers were determined by gel permeation chromatography (GPC). All measurements were performed on a Waters GPC system with a column of polysulfone styrenes. The GPC was equipped with differential refractive index and UV detectors. Polymer solutions (2 mg/mL) were prepared in DMSO, filtered (1 µm PTFE) and 100 µL of the solution were added to the column. The measurements were carried out at 80 °C. with a flow rate of 1 mL/min. Samples were read by a UV detector at a wavelength of 280 nm. Pullulan standards in DMSO with 0.1 M LiBr were used for calibration. All calculated masses were taken from the chromatograms presented in the Supplementary Materials.

#### 3.2.3. NMR Spectroscopy

All NMR spectra were measured on a Unity INOVA 500 NB spectrometer from Varian at 298 K. The residual protons of the respective solvent served as an internal standard for the calibration of the ^1^H-NMR spectra and the ^13^C signals for the ^13^C-NMR spectra. Coupling constants (J) are reported in Hertz. Abbreviations to denote the multiplicity of a particular signal are s (singlet), d (doublet), t (triplet), dd (double doublet), q (quartet), and m (multiplet). The polymer composition was determined by H-NMR. The aromatic protons were set in relation to the hydroxy group of the HEAA component. Proton spectra from monomers and polymers are also presented in the additional information.

#### 3.2.4. Dynamic Differential Calorimetry (DSC)

All measurements were carried out on a DSC 204 F1 Phoenix spectrometer from Netzsch (Leina Germany) under a nitrogen atmosphere. Aluminum crucibles were used as the crucible. A sample amount of 5–10 mg was weighed each time and double or triple cycle temperature programs in a temperature range from 50 to 250 °C. with a heating/cooling rate of 10 K/min were used. The glass transition temperature (T_g_), melting and crystallization temperature were determined during the second and third heating cycles. For pure substances, the onset temperature was used as the melting/crystallization temperature, and the peak maximum for formulations. The OmniCure S2000 (Göttingen, Germany) extension with a mercury lamp was used for photo-DSC studies. Sample amounts of 1–3 mg were weighed in an open aluminum crucible. The sample was tempered at 25 °C and the irradiance was 1 W/cm^2^.

#### 3.2.5. Adhesion Test

Cross-cut: The adhesion of the polymer coatings was measured according to DIN EN ISO 2409. Polymer coatings were prepared by spin coating (2500 rpm, 120 s, 1000 rpm, 30 s) with 5% polymer in DMSO onto cleaned glass substrates and dried for 60 min at 100 °C. A grid (6 × 1 mm) was cut into the polymer films using the cutting tool and the iso-adhesive tape was firmly fixed onto it. A grid (6 × 1 mm) was cut into the polymer films using the cutting tool. The iso-adhesive tape was firmly fixed on it and removed in a uniform peeling motion. This grid cut is evaluated by visual inspection using reference images. A distinction is made between cross-cut class 0 (good adhesion) and 5 (poor adhesion) (see Supplementary Materials).

Tensile shear test: Polycarbonates and titanium (Ti-6Al-4V, generatively manufactured by laser beam melting + blasted) with dimensions of 15 × 5 × 1 mm (15 × 3 × 1 mm for XMA_5_/PEGDA_0.1_) were used as substrates. The Titan substrates were washed in acetone and dried with compressed air. The protective film of the polycarbonate substrates was removed immediately before bonding. All bonding was performed on a bluepoint 4 ecocure UV point source from Dr. Hönle (Gilching, Germany) in a sealed chamber under an Argon atmosphere. A high-pressure mercury lamp with 150 W served as a radiation source. The full UV spectrum was used. The irradiation time was 300 s and the radiation intensity was set to 85%. The UV intensity at the sample location was determined to be 0.22 mW/cm^2^ at 240 nm and 14.9 mW/cm^2^ at 365 nm. The samples were measured in a small tensile shear machine at a speed of 5 × 10^−3^ mm s^−1^ at room temperature. The system allows a very accurate measurement of displacements via video extensometry. The adhesive layer thickness was 0.05 mm and the overlap length was 5 mm for glues with 5 mol% XMA and 3 mm for XMA_5_/PEGDA_0.1_.

### 3.3. Synthesis of Monomers

#### 3.3.1. Synthesis for *N*-(3,4-Dihydroxyphenyethyl) Acrylamide (DMA)

10 g of sodium borate and 4 g of NaHCO_3_ were stirred in 100 mL of degassed water under a nitrogen atmosphere. 5.00 g of dopamine hydrochloride (26.4 mmol) were added. 4.7 mL methacrylic anhydride (31.6 mmol) were separately diluted in 15 mL degassed THF and slowly added dropwise. The pH of the reaction mixture was monitored periodically with pH paper and maintained slightly basic (pH 8−9) with the addition of 1.0 M NaOH. The reaction was stirred at room temperature overnight. The reaction mixture was filtered and the precipitate was washed with ethyl acetate. The filtrate was acidified to pH 2 with concentrated HCl. The organic layer was separated, and the aqueous phase was extracted with EtOAc (3 × 50 mL), dried over MgSO_4_, and the solvent was evaporated under reduced pressure. The crude product was purified by column chromatography on silica gel with a mixture of DCM/MeOH (9:1) as eluent. A solid was obtained (yield: 3.86 g, 17.4 mmol, 66%) ^1^H-NMR (500 MHz, DMSO-d_6_) δ = 8.76 (s, 1H), 8.64 (s, 1H,). 7.94 (t, J = 5.7 Hz, 1H). 6.63 (d, J = 7.9 Hz, 1H). 6.58 (d, J = 2.2 Hz, 1H). 6.43 (dd, J = 7.9, 2.2 Hz, 1H). 5.62 (s, 1H). 5.29 (s, 1H). 3.23 (dt, J = 9.0, 5.9 Hz, 1H). 2.56 (t, J = 7.6 Hz, 2H). 1.84 (s, 3H). APT-NMR (126 MHz, DMSO-d_6_) δ = 167.3, 145.1, 143.5, 140.1, 130.3, 119.2, 118.8, 116.0, 115.5, 41.0, 34.6, 18.7.

#### 3.3.2. Synthesis for *N*-(3,4-Dihydroxyphenyethyl) Acrylamide (DA)

Sodium borate decahydrate (25.2 g, 66 mmol), dopamine hydrochloride (25.0 g, 134 mmol), and Na_2_CO_3_ (21.0 g, 198 mmol) were stirred in an ice bath under a nitrogen atmosphere in 200 mL of degassed water. A solution of acryloyl chloride (12.1 g, 10.9 mL 134 mmol) in 25 mL degassed THF was slowly added dropwise to this suspension. After the addition of acryloyl chloride was complete, a second portion of Na_2_CO_3_ (14.0 g, 132 mmol) was added. The pH of the solution was kept at (pH = 9) with 1 M NaOH. The suspension was stirred at room temperature overnight. The suspension was acidified to pH 1 with concentrated HCl. The precipitate was filtered off and washed with ethyl acetate. The organic layer was separated, and the aqueous phase was extracted with EtOAc (3× 50 mL), dried over MgSO_4_, and the solvent was evaporated under reduced pressure. The crude product was purified by column chromatography on silica gel with a mixture of DCM/MeOH (9:1) as eluent. A solid was obtained (yield: 23.9 g, 115 mmol, 86%). ^1^H-NMR (500 MHz, DMSO-d_6_) δ = 8.77 (s, 1H), 8.67 (s, 1H), 8.14 (t, J = 5.4 Hz, 2H), 6.64 (d, J = 7.9 Hz, 1H), 6.59 (s, 1H), 6.47–6.41 (m, 1H), 6.20 (dd, J = 17.1, 10.1 Hz, 1H), 6.07 (dd, J = 17.1, 1.9 Hz, 1H), 5.56 (dd, J = 10.1, 1.9 Hz, 1H), 3.27 (q, J = 6.6 Hz, 2H), 2.56 (t, J = 7.4 Hz, 2H). ^13^C-NMR (126 MHz, DMSO-d_6_) δ 167.3, 145.1, 143.6, 131.9, 130.3, 129.6, 125.1, 119.3, 116.1, 115.6, 40.7, 34.7.

#### 3.3.3. Synthesis for *N*-Phenethyl Methacrylamide (PMA)

Phenylethylamine (5.51 g, 45.5 mmol) and triethylamine (6.9 mL, 49.5 mmol) were dissolved in 250 mL DCM. Methacrylic anhydride (16.8 mL, 105.3 mmol) was then slowly added at 0 °C. After the addition, the reaction solution was stirred further at RT overnight. The solvent was removed under reduced pressure and the residue was purified by column chromatography (DCM/MeOH, 99/1). A yellow oil was obtained as product (7.52 g, 39.7 mmol, 87.4%). ^1^H-NMR (500 MHz, CDCl_3_) δ = 7.52–7.02 (m, 5H), 5.84 (br, 1H), 5.63 (s, 1H), 5.30 (s,1H), 3.59 (q, J = 6.9, 2H), 2.88 (t, J = 7.0, 2H), 1.94 (s, 3H). ^13^C-NMR (126 MHz, CD_2_Cl_2_) δ = 168.6, 141.0, 139.8, 129.3, 129.1, 126.9, 119.3, 41.3, 36.2, 18.9.

#### 3.3.4. Synthesis for *N*-(4-Hydroxyphenethyl) Methacrylamide (TMA)

Methacrylic anhydride (11.4 mL, 76.5 mmol) was added dropwise to a solution of tyramine (10.0 g, 72.9 mmol) in 360 mL of dry MeOH and the mixture was stirred at room temperature overnight. The solvent was removed under reduced pressure and the residue was purified by column chromatography (DCM/MeOH 9: 1). A colorless solid was obtained as product (11.2 g, 54.6 mmol, 74.8%). ^1^H-NMR (500 MHz, DMSO-d_6_) δ = 9.18 (s, 1H), 7.95 (t, J = 5.3, 2H), 6.98 (d, J = 8.4, 2H), 6.68 (d, J = 8.4, 2H), 5.61 (s, 1H), 5.29 (s, 2H), 3.25 (dt, J = 5.3, 6.7, 2H), 2.63 (t, J = 6.7, 2H), 1.84 (s, 3H). ^13^C-NMR (126 MHz, DMSO-d_6_) δ = 167.4, 155.6, 140.1, 129.6, 129.5, 118.8, 115.1, 41.0, 34.3, 18.7.

### 3.4. General Procedure for the Synthesis of Polymers

Acrylamide monomers (DMA, DA, PMA, and TMA) and AIBN (1%) were dissolved in dry DMF in a multi-necked flask under a nitrogen atmosphere. The appropriate amount of *N*-hydroxyethyl acrylamide was added as a DMF solution via a cannula syringe. The total monomer concentration was 0.1 mol/L. The solution was stirred at 75 °C for 20 h. The reaction mixture was cooled to room temperature and concentrated on a rotary evaporator. The residue was dissolved in a small amount of methanol and precipitated in diethyl ether. The precipitated product was filtered off with suction. The precipitation was repeated. The product was dried at 40 °C. in a vacuum cabinet. The products were obtained as colorless solids. For the polymer P(HEAA/DMA_10_) acrylamide monomer DMA (212.2 mg, 959 μmol, 10 mol%), HEAA (990.0 mg, 8.60 mmol, 90 mol%) and AIBN (16.1 mg, 98.0 µmol, 1 mol%) polymerized in 95 mL DMF.

P(HEAA/DMA): ^1^H-NMR (500 MHz, DMSO-d_6_) δ = 8.77 (OH), 8.62 (OH), 7.84–7.15 (NH), 6.63 (aromatic), 6.59 (aromatic), 6.44 (aromatic), 5.11–4.58 (OH), 3.42 (aliphatic), 3.36 (aliphatic), 3.30–2.85 (aliphatic), 2.14–1.80 (backbone), 1.71–1.03 (backbone), 1.04–0.64 (backbone).

P(HEAA/DA): ^1^H-NMR (500 MHz, DMSO-d_6_) δ = 8.96–8.32 (OH), 8.02–7.03 (NH), 6.73–6.50 (aromatic), 6.42 (aromatic), 5.20–4.51 (OH), 3.58–3.31 (aliphatic), 3.33–2.87 (aliphatic), 2.21–1.72 (backbone), 1.72–1.16 (backbone).

P(HEAA/DMA): ^1^H-NMR (500 MHz, DMSO-d_6_) δ = 7.97–7.36 (NH), 7.35–7.06 (aromatic), 5.14–4.58 (OH), 3.62–3.29 (aliphatic), 3.29–2.85 (aliphatic), 2.77–2.58 (backbone), 2.32–1.74 (backbone), 1.73–1.15 (backbone), 1.02–0.72 (backbone).

P(HEAA/TMA): ^1^H-NMR (500 MHz, DMSO-d_6_) δ = 9.16 (OH), 8.02–7.22 (NH), 6.97 (aromatic), 6.67 (aromatic), 5.26–4.47 (OH), 3.84–3.25 (aliphatic), 3.25–2.77 (aliphatic), 2.68–2.24, 2.20–1.72 (backbone), 1.74–1.17 (backbone), 1.03–0.66 (backbone).

## 4. Conclusions

In summary, we have described solvent-based polymerizations from easily prepared acrylamides. We synthesized new adhesive materials from catechol-containing acrylamides with HEAA as the literature-known antifouling component. Copolymers could be successfully made from all monomers. The proportions of monomers varied between 30–80 mol% per monomer, while the homopolymers were not suitable for synthesis. The acrylamide-modified dopamine monomer is significantly more reactive than the methacrylic-modified monomer. The higher reactivity has been demonstrated with the formation of P(HEAA/DA_80_). Copolymers with Catechol-functionality were obtained up to a molar content of 30 mol% for the meth acrylamide monomer DMA and even 80 mol% for the acrylamide monomer DA without protecting groups. An adhesion test DIN EN ISO 2409 showed very good adhesion to glass for all polymers. The same monomers are suitable as components in photoreactive adhesives, which were comparatively investigated on titanium samples in tensile tests. We were able to confirm the adhesive effect caused by the catechol function. We demonstrated that catechol-containing photopolymers DMA_5_/PEGDA_0.1_ exhibit stronger adhesion to polycarbonate and titanium than equivalents without catechol function.

## Figures and Tables

**Table 1:** Experimental data of the synthesized acrylamide copolymers.

	DA/DMA/TMA/PMA Monomer_feed_ mol%	DA/DMA/TMA/PMA Monomer_found_		Yield %	M_n_ kg/mol	DP	M_w_ kg/mol	PDI	T_g_ °C	Cross-Cut Class
		mol%	wt%							
P(HEAA)	100	-	100	51	2.5	22	5.7	2.3	176.0	0
P(HEAA/DA_10_)	10	9	15	69	2.4	19	4.8	2.0	n. d.	0
P(HEAA/DA_30_)	30	29	42	47	3.7	21	7.2	1.9	123.4	0
P(HEAA/DA_50_)	50	50	64	40	3.2	20	5.4	1.7	127.9	0
P(HEAA/DA_80_)	80	80	88	60	3.7	20	7.8	2.1	128.9	n. a.
P(DA)	100	-	-	0	no polymerization					-
P(HEAA/TMA_10_)	10	10	17	82	3.1	25	6.6	2.1	111.3	0
P(HEAA/TMA_30_)	30	28	41	63	3.4	24	7.5	2.2	122.7	0
P(HEAA/TMA_50_)	50	50	64	56	4.5	28	12.4	2.7	128.2	0
P(HEAA/TMA_80_)	80	-	-	0	no polymerization					-
P(TMA)	100	-	-	0	no polymerization					-
P(HEAA/DMA_10_)	10	12	21	56	11.0	87	30.1	2.7	131.1	0
P(HEAA/DMA_20_)	20	23	36	42	3.8	28	10.2	2.7	126.3	0
P(HEAA/DMA_30_)	30	33	49	53	3.8	23	10.2	2.0	126.0	0
P(HEAA/DMA_50_)	50	-	-	0	no polymerization					-
P(HEAA/DMA_67_)	67	-	-	0	no polymerization					-
P(HEAA/DMA_80_)	80	-	-	0	no polymerization					-
P(DMA)	100	-	-	0	no polymerization					-
P(HEAA/PMA_10_)	10	9	14	69	2.7	22	5.4	2.0	110.5	0
P(HEAA/PMA_20_)	20	20	29	63	2.5	19	4.4	1.8	99.9	0
P(HEAA/PMA_30_)	30	31	42	57	2.9	21	5.8	2.0	110.5	0
P(HEAA/PMA_50_)	50	-	-	0	no polymerization					-

The molar ratio of the polymer composition was determined by ^1^H-NMR spectroscopy. M_n_ (average molecular weights), DP (degree of polymerization—the number of monomeric units in a polymer), M_w_ (molecular weight distributions), and PDI (polydispersity index) of the polymers were determined by gel permeation chromatography (GPC). The glass transition temperature (T_g_) was determined by dynamic differential calorimetry (DSC), and the cross-cut class was measured according to DIN EN ISO 2409 with 0 (good adhesion) and 5 (poor adhesion).

**Table 2:** Results of photo-DSC and tensile shear test: Rp_max_ (maximum photo-curing rate), t_max_: (time to reach Rp_max_), ΔH_max_ (maximum generated photo-curing heat), DBC_max_ (maximum double bond conversion), F_max_ (maximum force at break).

Glue Formulation with 5 mol% of	Rp_max_ s^−1^	t_max_ s	ΔH_max_ J	DBC_max_ %	F_max_ MPa	Elongation_max_ %
PMA	0.49	12.0	−646.2	91. 9	1.26	0.46
TMA	0.29	6.0	−610.2	93.2	1.68	1.00
DMA	0.47	3.6	−516.1	75.4	0.66	1.98
DA	0.53	4.8	−479.6	70.0	n.a	n.a
PMA_5_/PEGDA_0.1_	0.68	4.8	−504.6	83.8	5.03	1.27
TMA_5_/PEGDA_0.1_	0.51	6.0	−582.8	94.8	6.02	1.57
DMA_5_/PEGDA_0.1_	0.50	3.6	−524.5	87.3	6.23	1.81

## Data Availability

See supplementary materials.

## Supplementary Materials

The following supporting information can be downloaded at: https://www.mdpi.com/article/10.3390/molecules27134027/s1. Figures S1–S21, ^1^H-NMR spectra of Homo and Co-Polymers.

## Sample Availability

Samples of the compounds are not available from the authors.

## Abbreviations

DA	*N*-(3,4-dihydroxyphenyethyl) acrylamide
DMA	*N*-(3,4-dihydroxyphenyethyl) meth acrylamide
HEAA	*N*-(2-hydroxyethyl) acrylamide
PC	polycarbonate
PEGDA	poly(ethylene glycol) diacrylate
PMA	*N*-phenethyl methacrylamide
TMA	*N*-(4-hydroxyphenethyl) methacrylamide
XMA	DA, DMA, PMA and TMA
