# Supplementary material for: Synthesis and Characterization of Catechol-Containing Polyacrylamides with Adhesive Properties
Source: Molecules. 2022 Jun 23;27(13):4027. doi: 10.3390/molecules27134027 (PMC9268726; doi:10.3390/molecules27134027)
Supplement: Supplementary file 1 [file molecules-27-04027-s001.zip › molecules-1779393-supplementary.pdf]

Supplementary Materials

Synthesis and characterization of catechol-containing polyacrylamides

Kathleen Hennig[1], Wolfdietrich Meyer [1,*]

[1] Fraunhofer-Institut Angewandte Polymerforschung IAP, wolfdietrich.meyer@iap.fraunhofer.de

*Correspondence: kathleen.hennig@iap.fraunhofer.de, wolfdietrich.meyer@iap.fraunhofer.de

^{1}H-NMR spectra of Homo and Co-Polymers are given in the following figures. The mol content of modified monomers in the copolymers were calculated from peak a and peak b and calculated as follow:

$$A = \frac{\frac{a(\delta=6.5\ ppm)}{number\ of\ protons}}{\left(\frac{a(\delta=6.5\ ppm)}{number\ of\ protons} + b(\delta=5.0\ ppm)\right)} \cdot 100\ [\%]$$

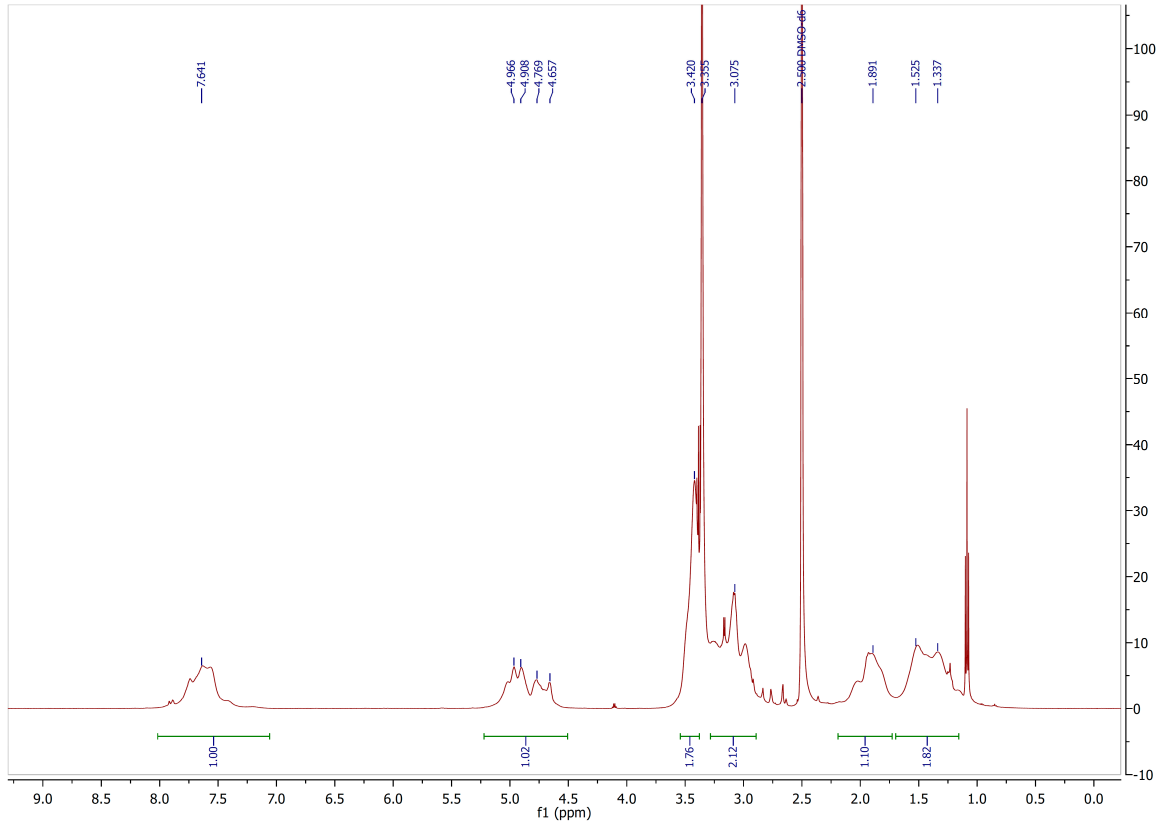

Figure S1. ^{1}H-NMR spectrum of P(HEAA) (DMSO-d$_6$, 500 MHz)

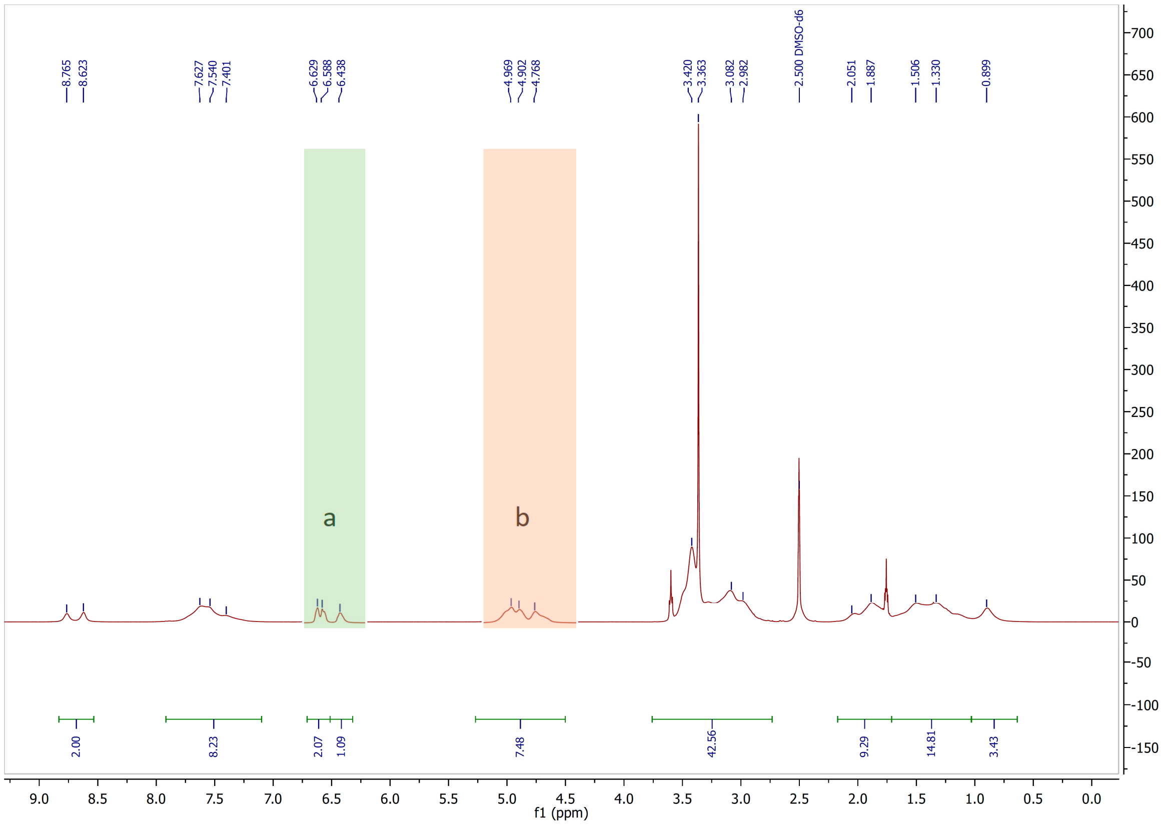

Figure S2. ^{1}H-NMR spectrum of P(HEAA/DMA$_{10}$) (DMSO-d$_6$, 500 MHz)

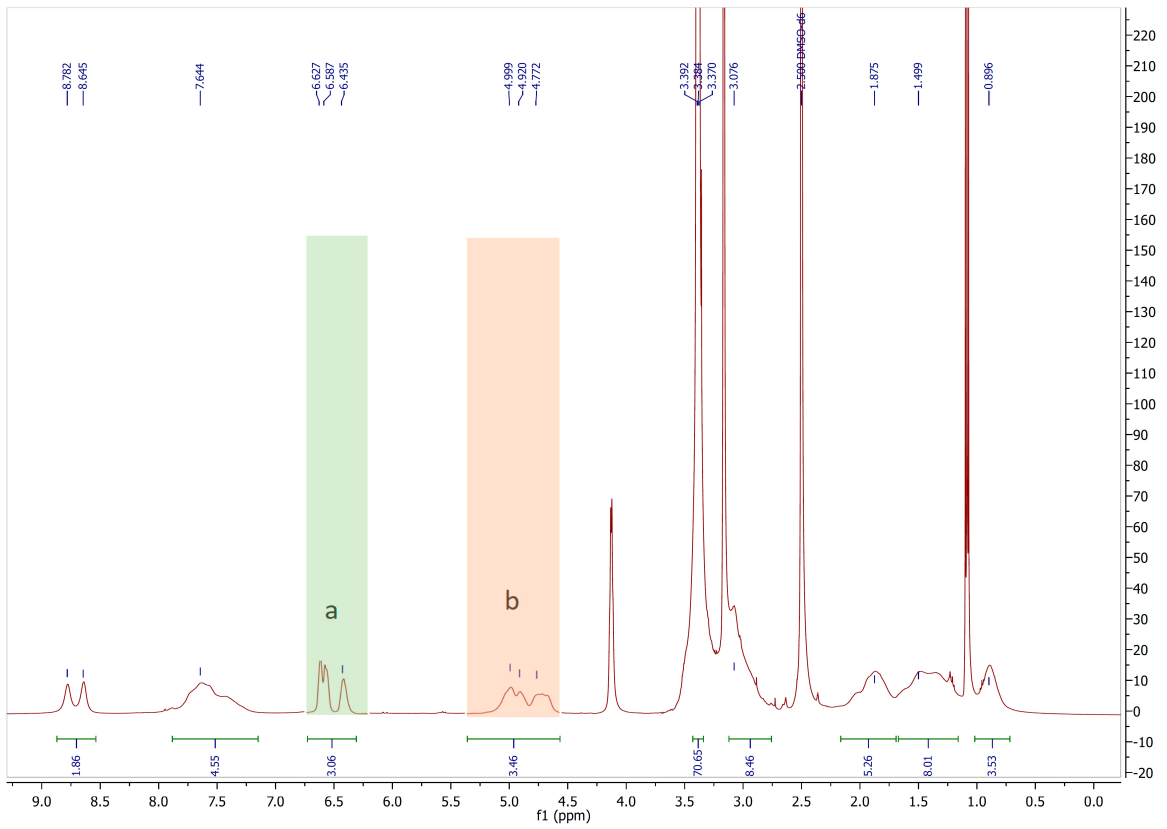

Figure S3. ^{1}H-NMR spectrum of P(HEAA/DMA$_{20}$) (DMSO-d$_6$, 500 MHz)

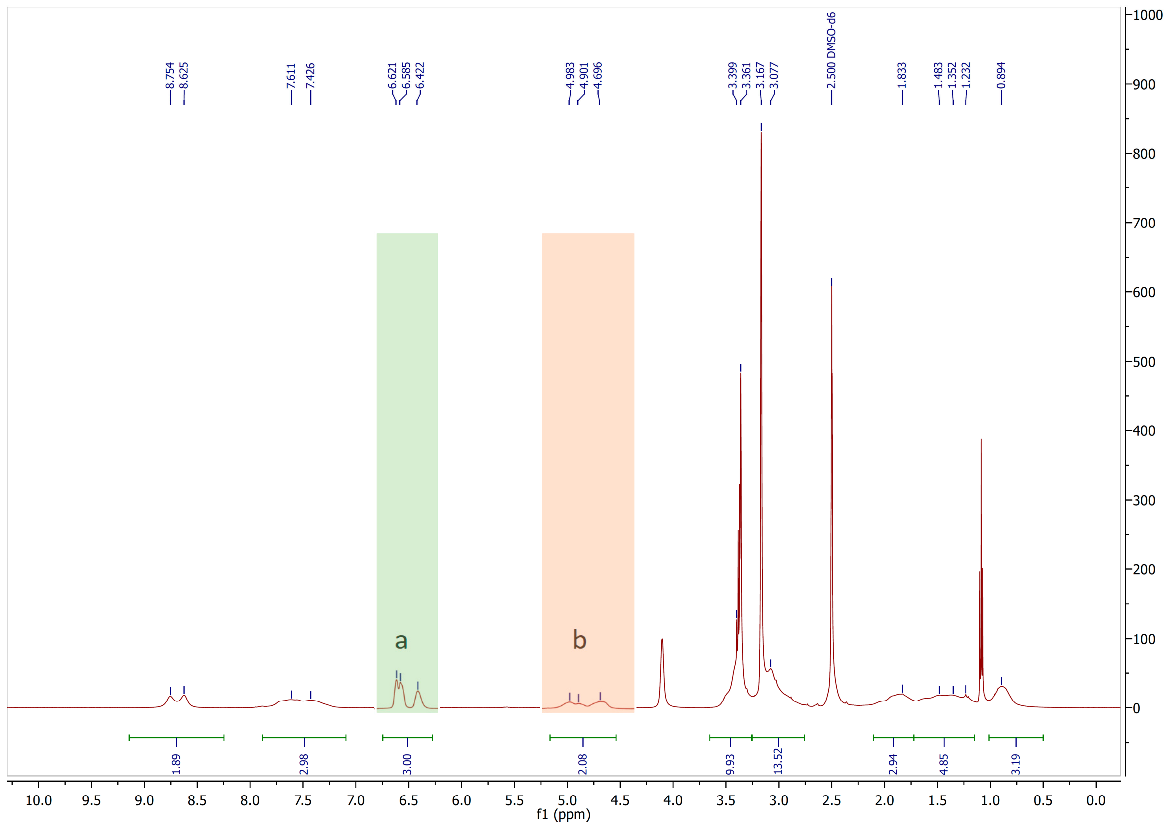

Figure S4. ^{1}H-NMR spectrum of P(HEAA/DMA$_{30}$) (DMSO-d$_6$, 500 MHz)

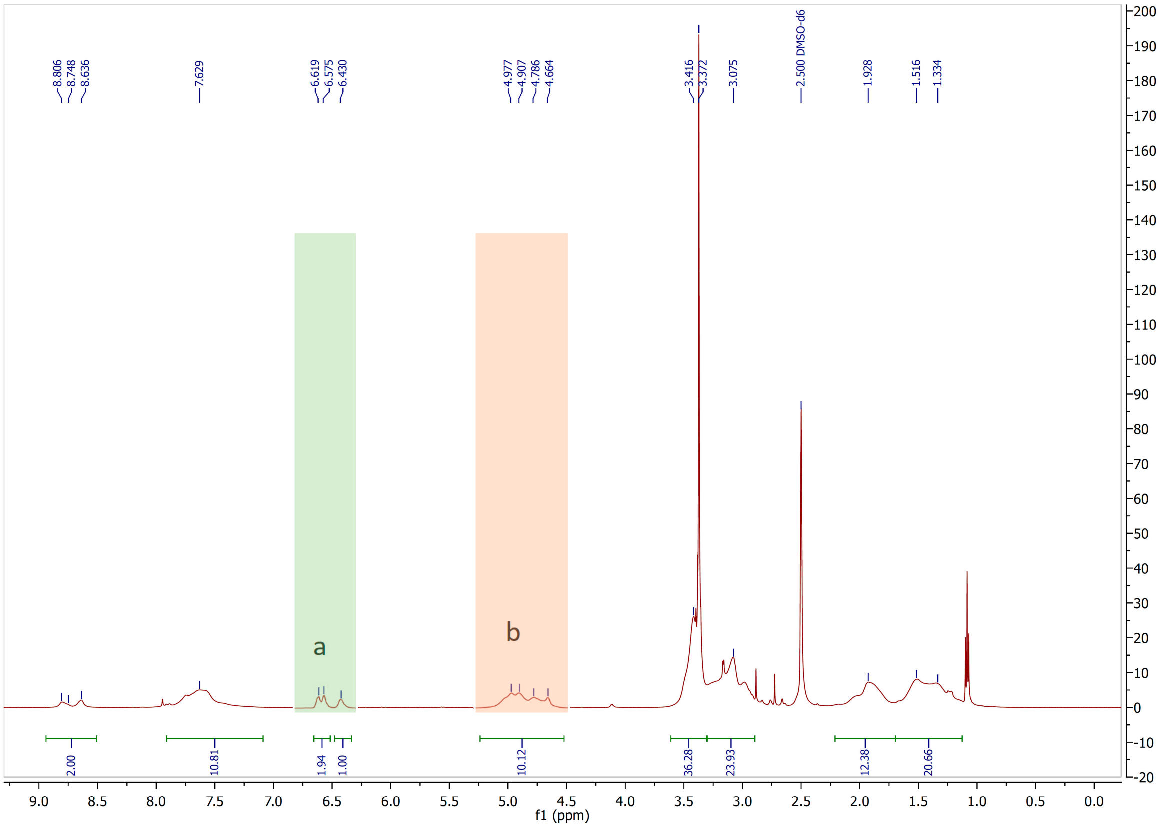

Figure S5. ^{1}H-NMR spectrum of P(HEAA/DA$_{10}$) (DMSO-d$_6$, 500 MHz)

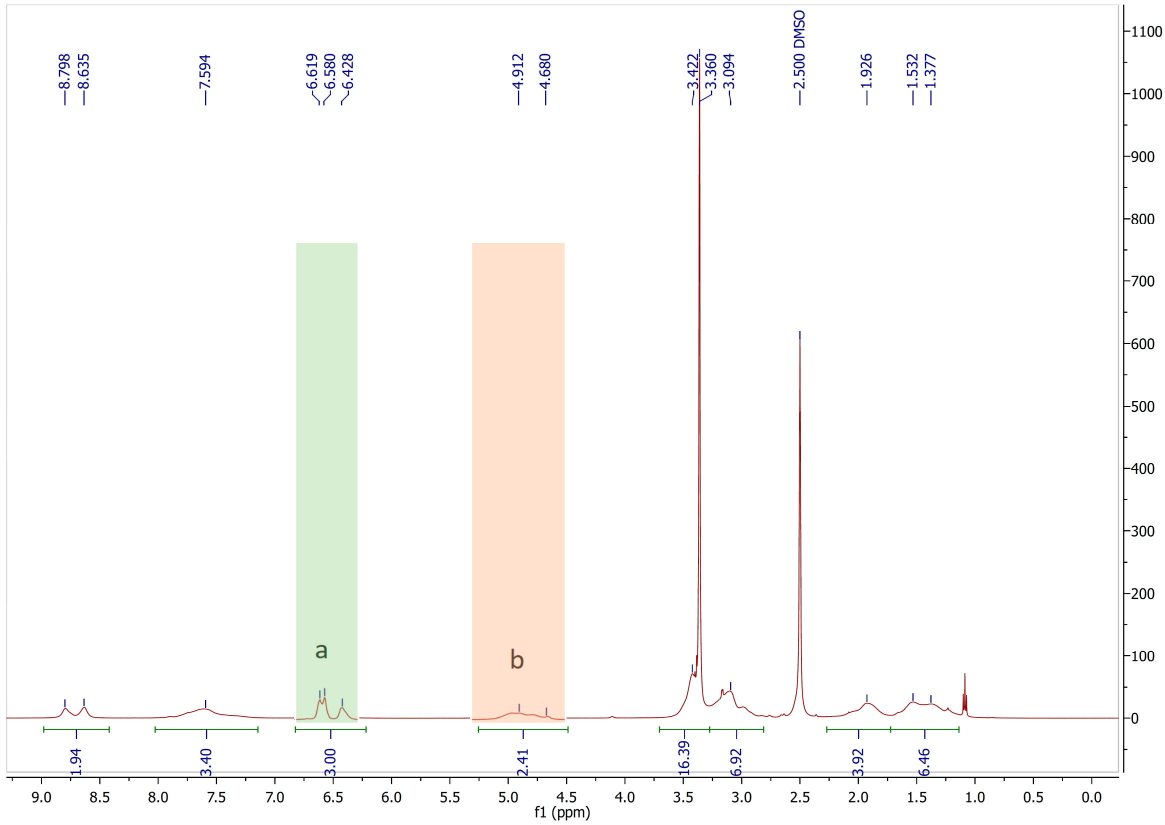

Figure S6. ^{1}H-NMR spectrum of P(HEAA/DA$_{30}$) (DMSO-d$_6$, 500 MHz)

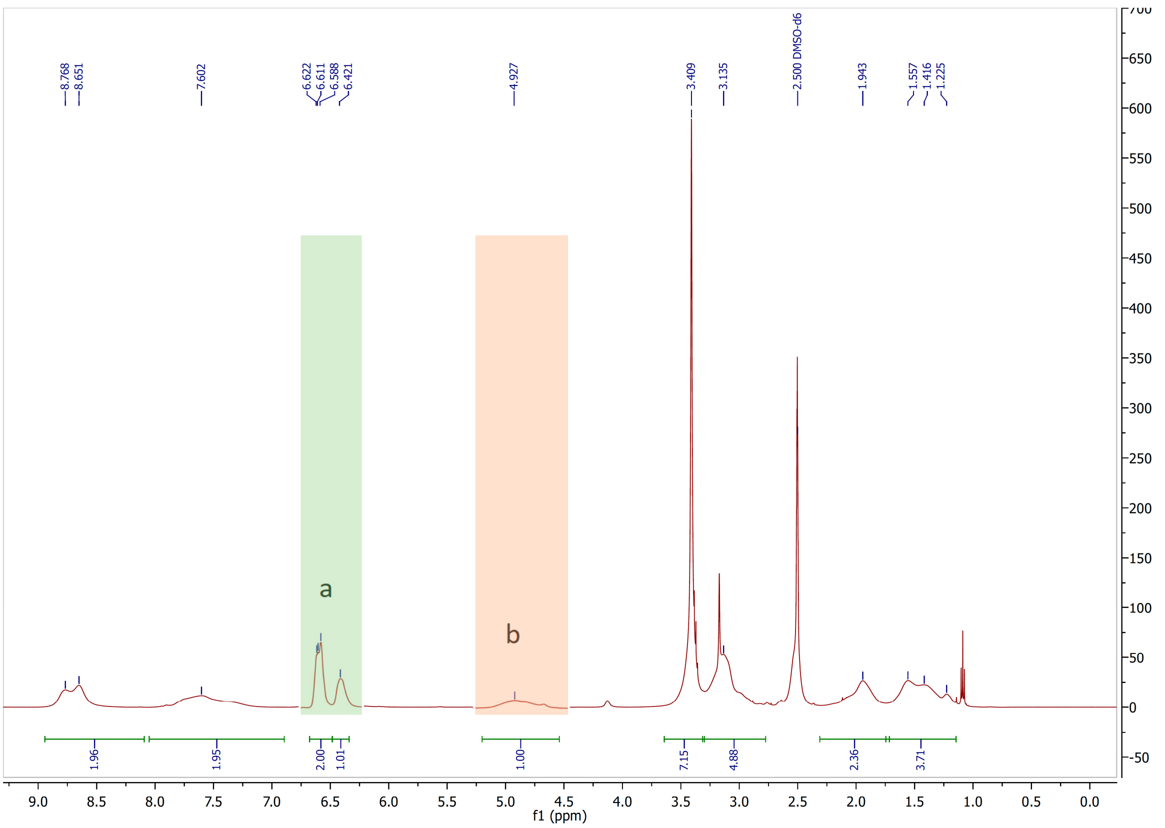

Figure S7. ^{1}H-NMR spectrum of P(HEAA/DA$_{50}$) (DMSO-d$_6$, 500 MHz)

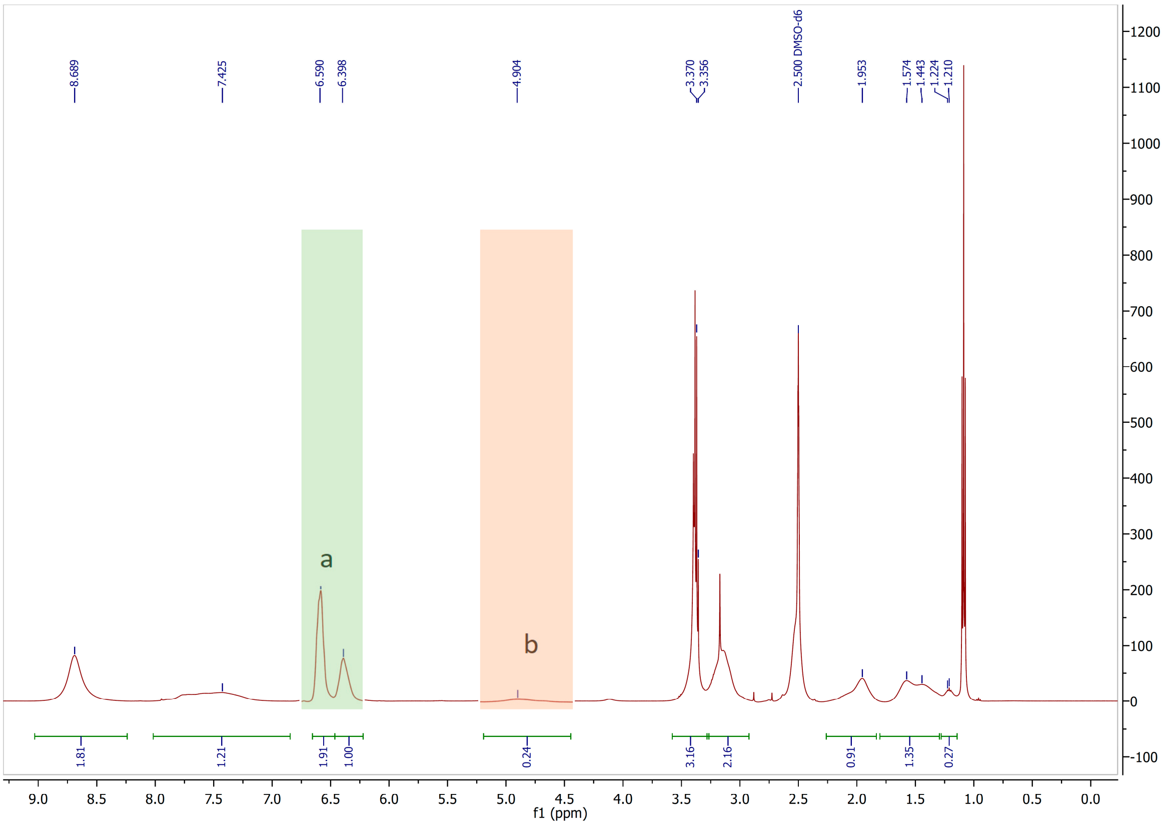

Figure S8. ^{1}H-NMR spectrum of P(HEAA/DA$_{80}$) (DMSO-d$_6$, 500 MHz)

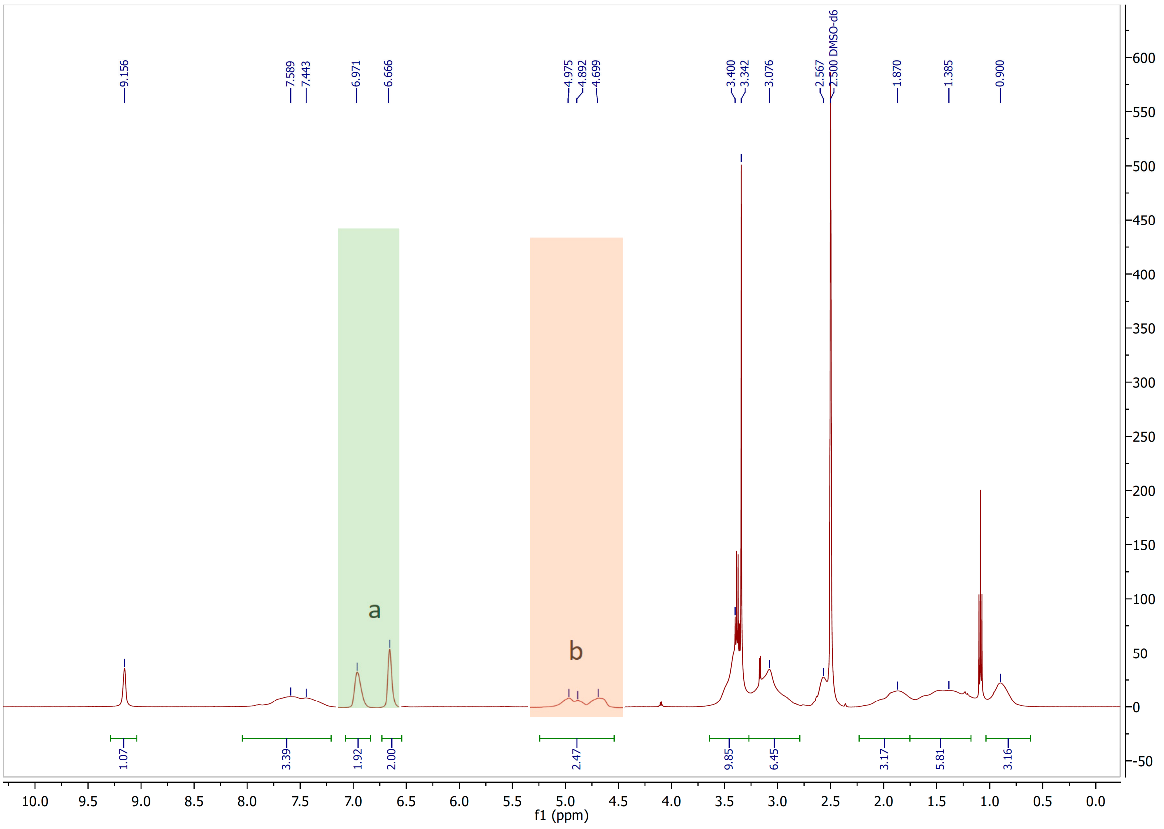

Figure S9. ^{1}H-NMR spectrum of P(HEAA/TMA$_{30}$) (DMSO-d$_6$, 500 MHz)

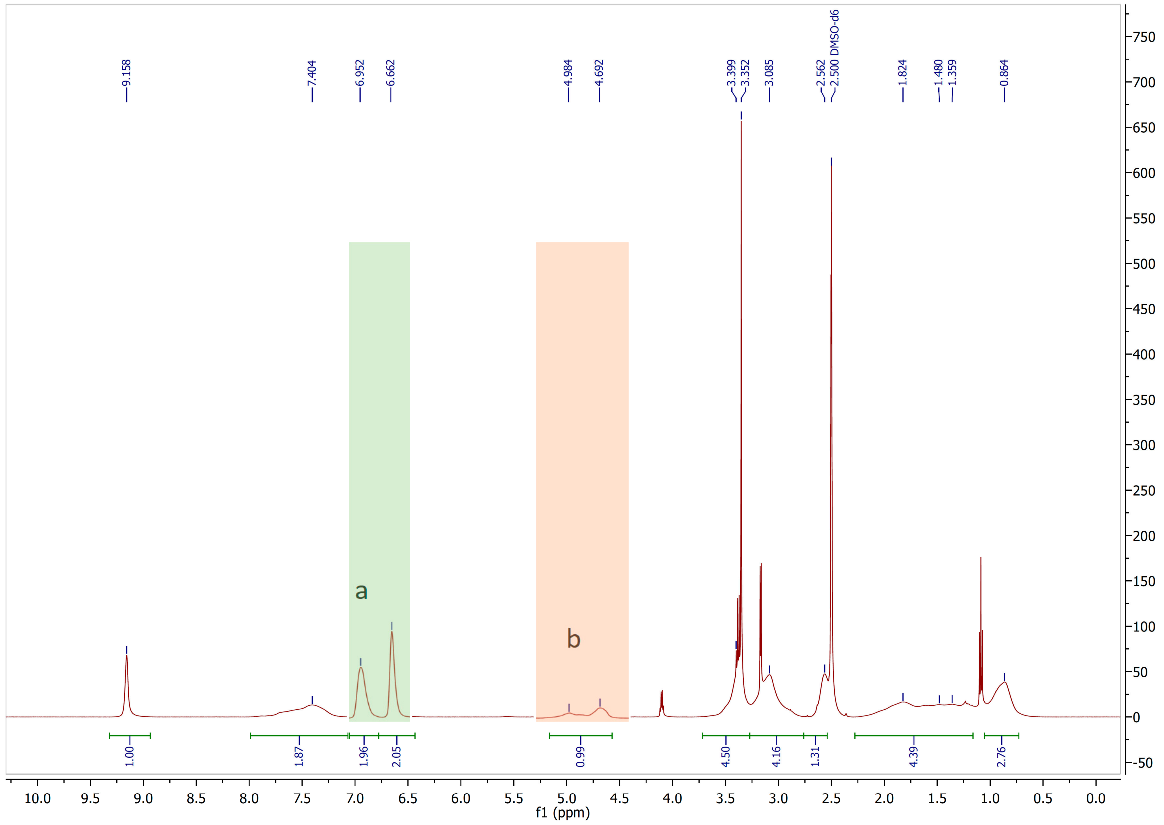

Figure S10. ^{1}H-NMR spectrum of P(HEAA/TMA$_{50}$) (DMSO-d$_6$, 500 MHz)

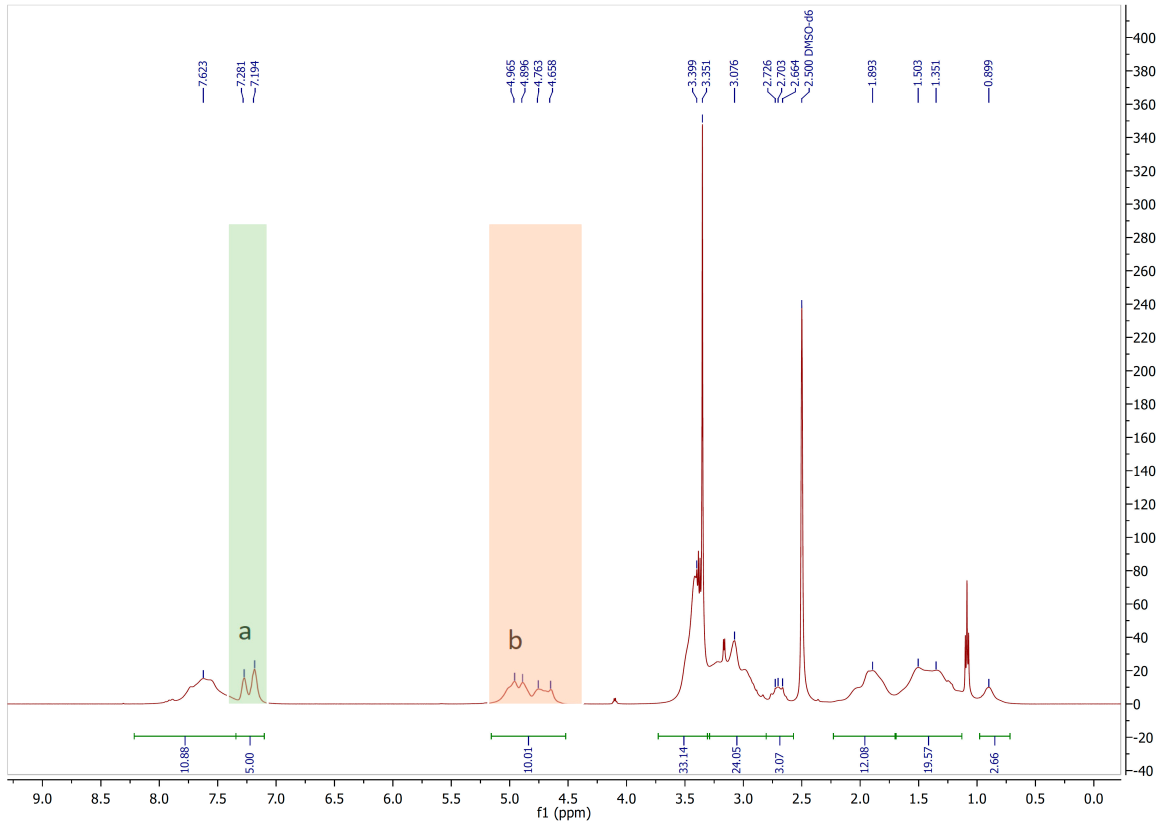

Figure S11. ^{1}H-NMR spectrum of P(HEAA/PMA$_{10}$) (DMSO-d$_6$, 500 MHz)

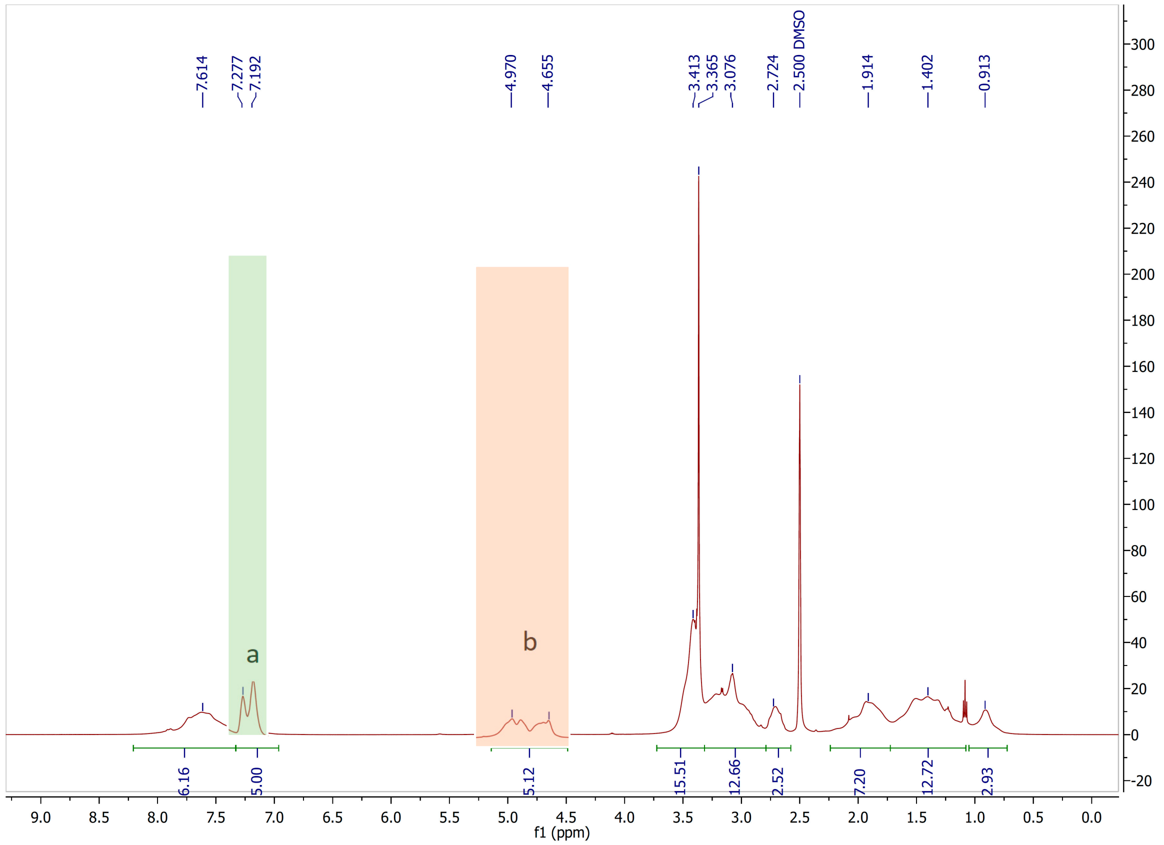

Figure S12. ^{1}H-NMR spectrum of P(HEAA/PMA$_{20}$) (DMSO-d$_6$, 500 MHz)

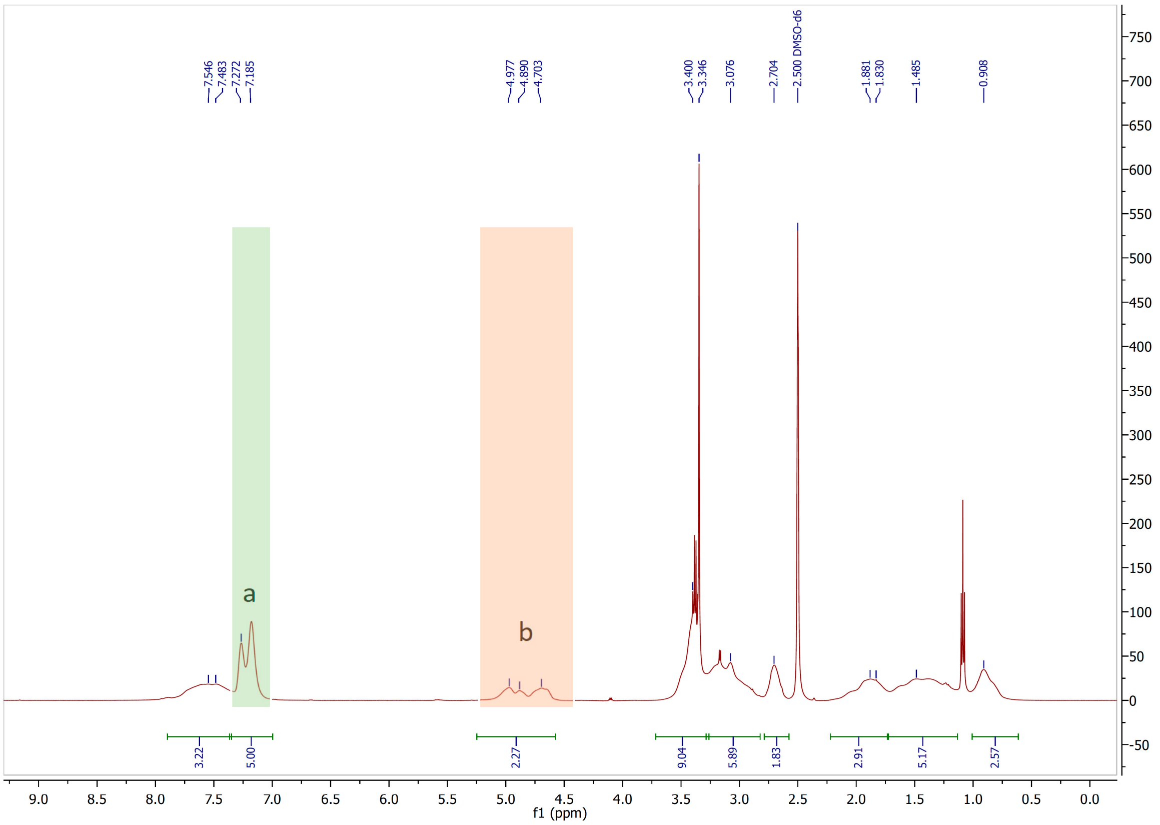

Figure S13. ¹H-NMR spectrum of P(HEAA/PMA$_{30}$) (DMSO-d$_6$, 500 MHz)

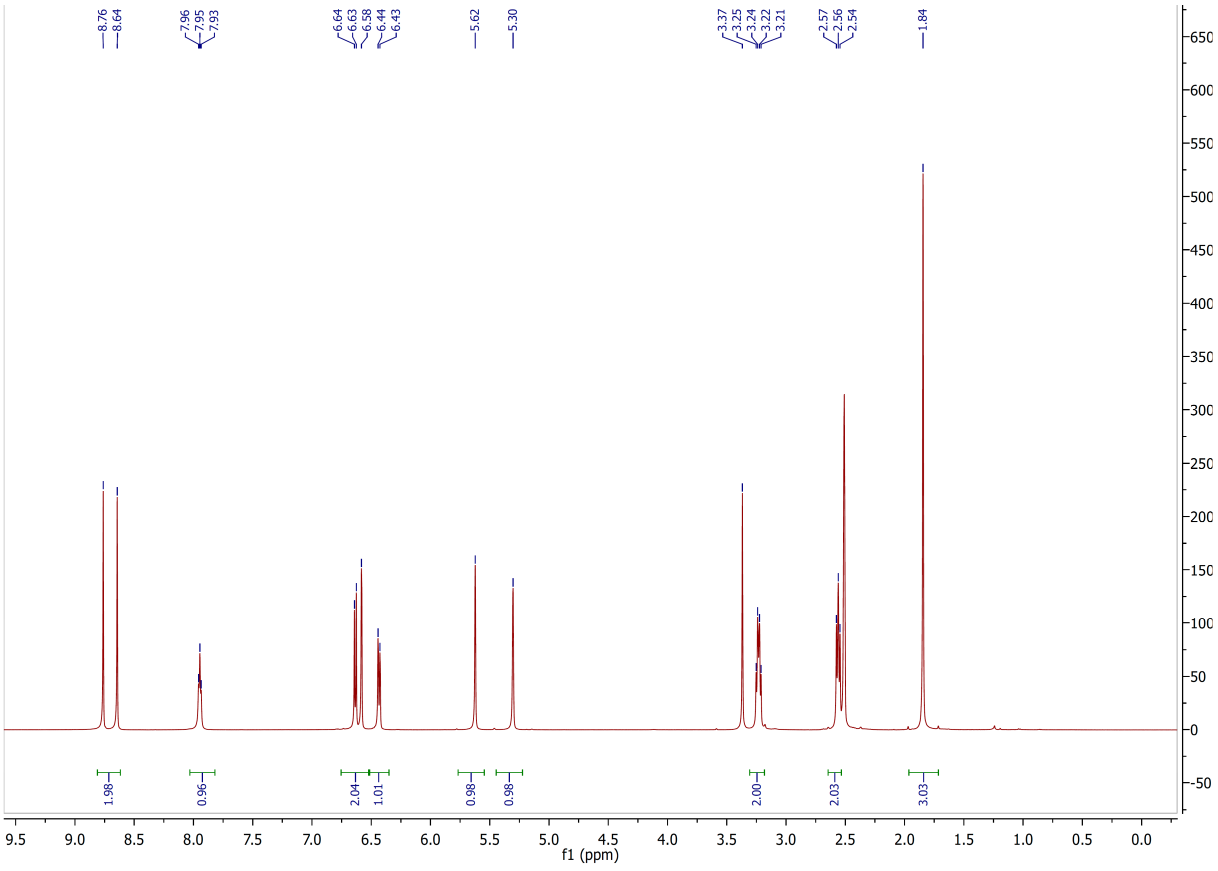

Figure S14: ¹H-NMR(DMA) (DMSO-d6, 500 MHz)

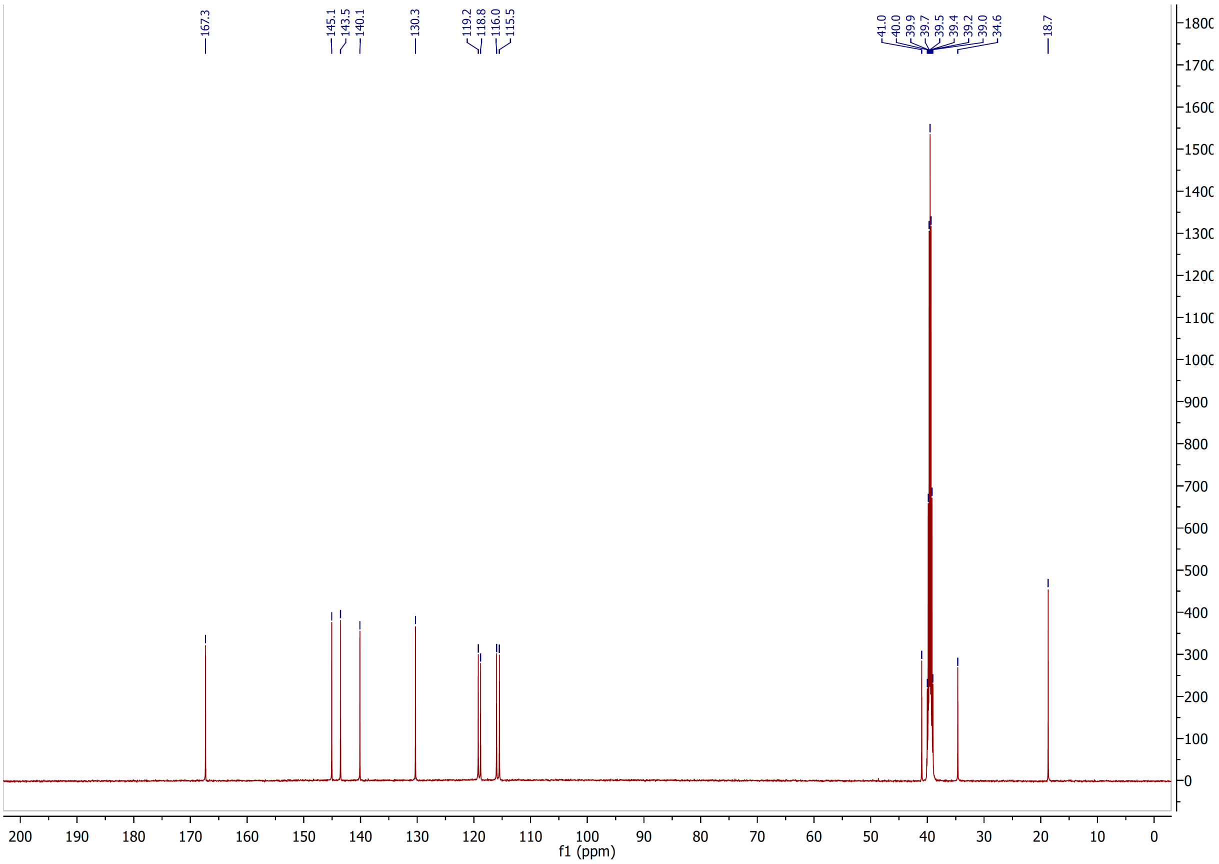

Figure S15: ^{13}C-NMR(DMA) (DMSO-d6, 126 MHz)

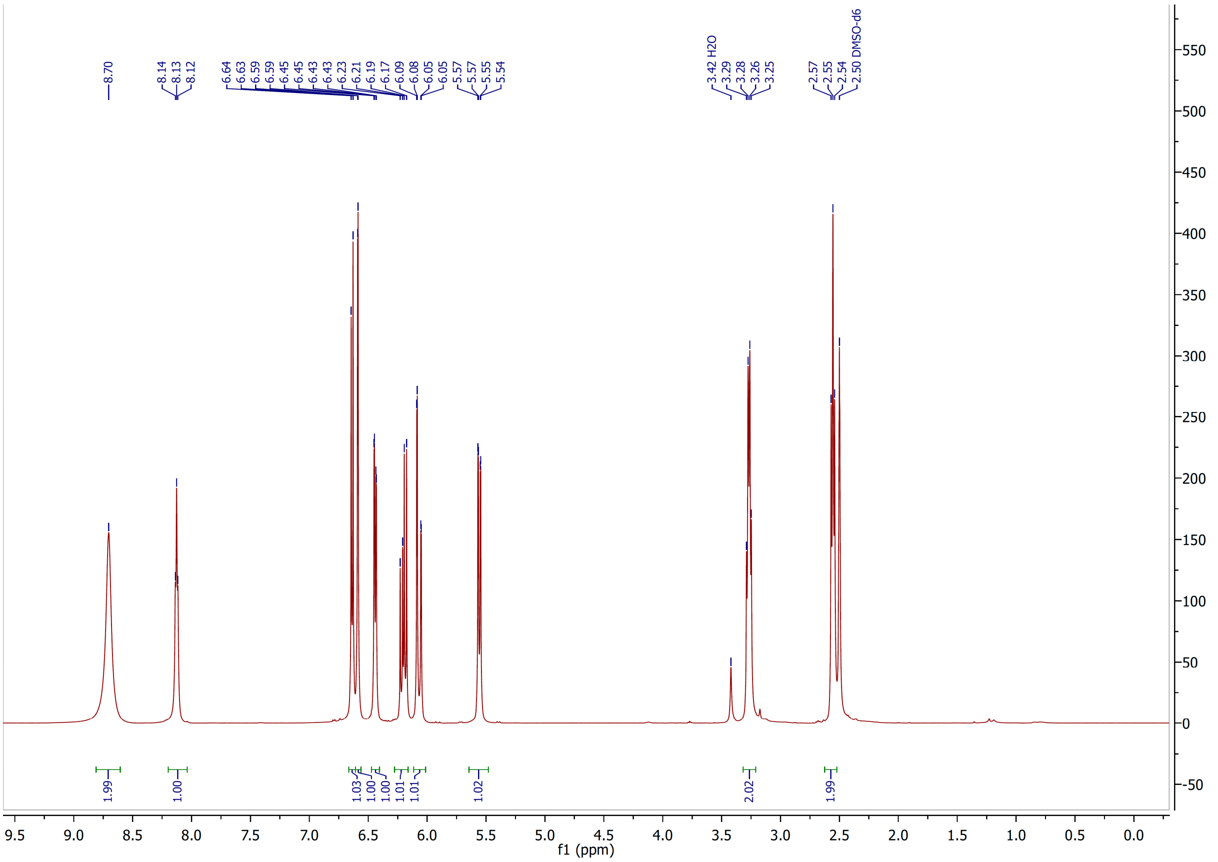

Figure S16: ^{1}H-NMR(DA) (DMSO-d6, 500 MHz)

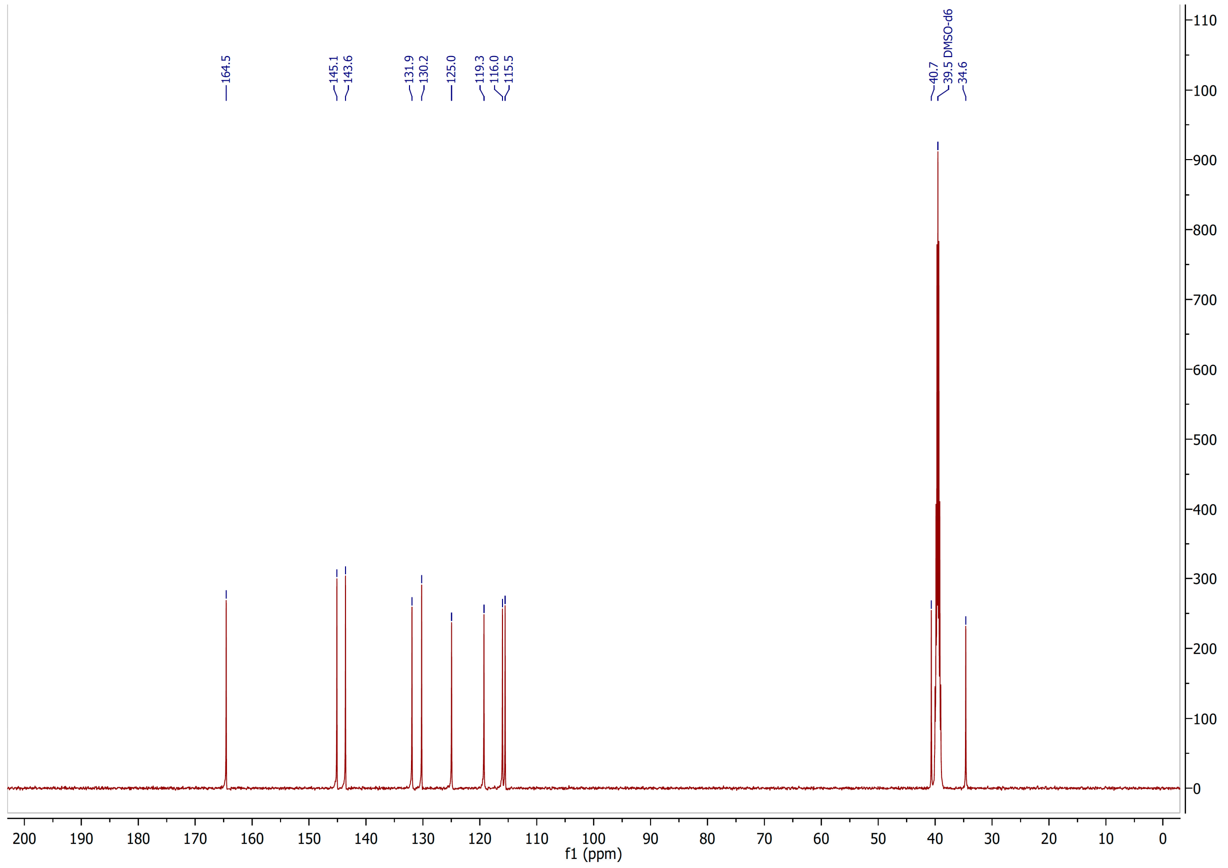

Figure S17: ^{13}C-NMR(DA) (DMSO-d6, 126 MHz)

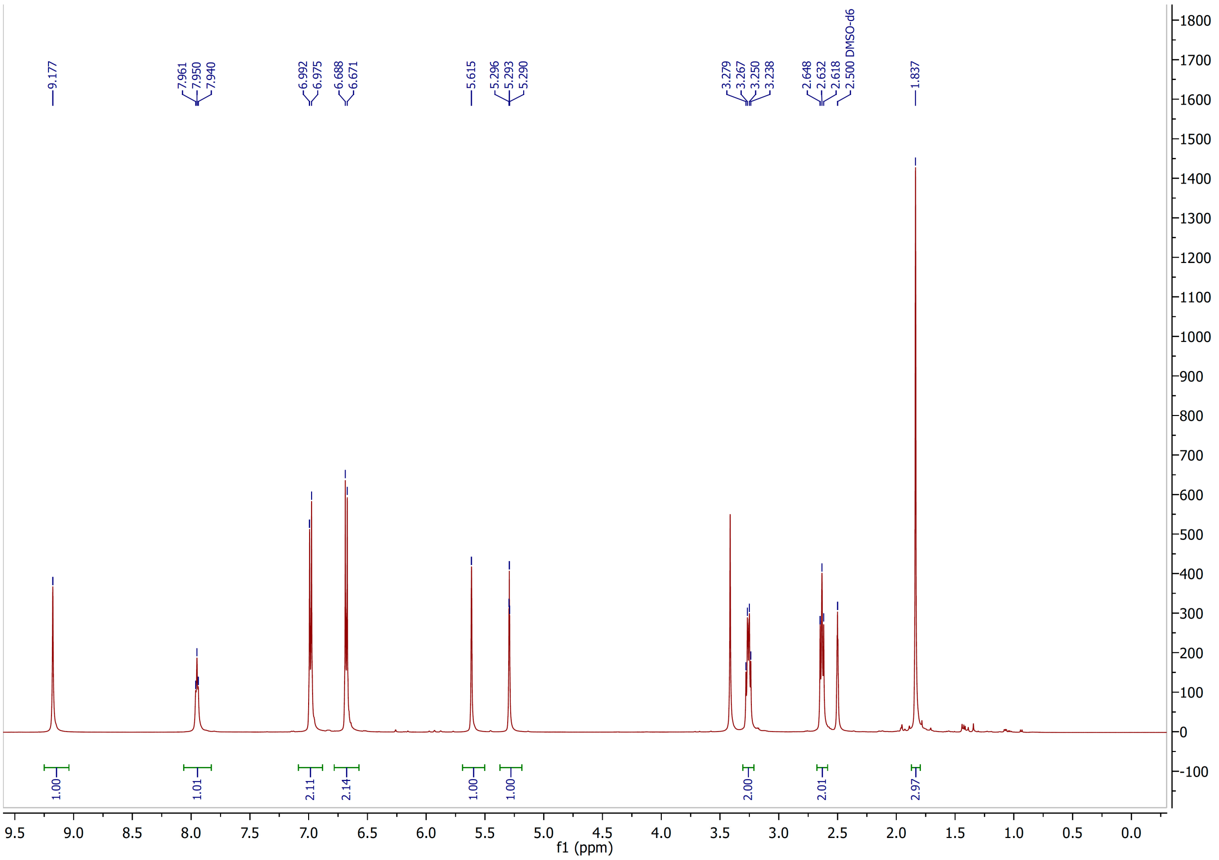

Figure S18: ^{1}H-NMR(TMA) (DMSO-d6, 500 MHz)

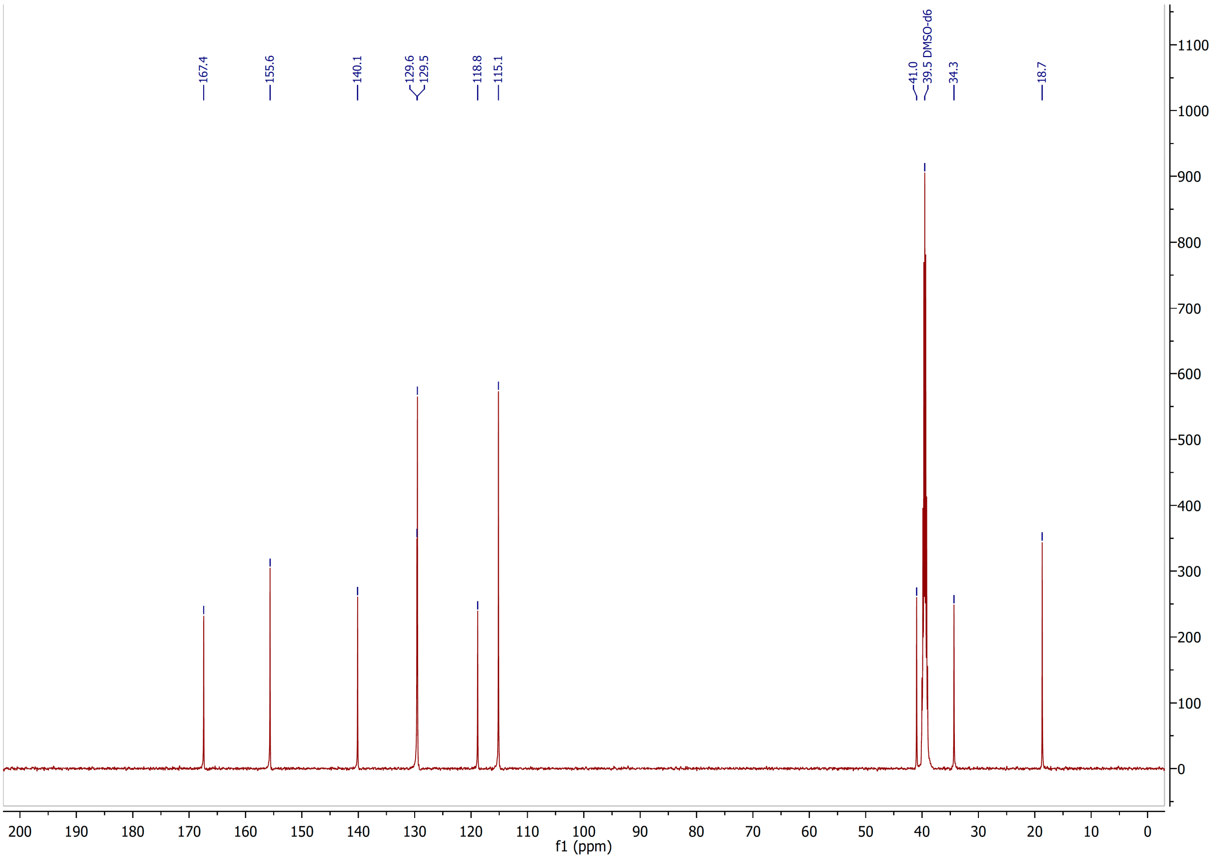

Figure S19: ^{13}C-NMR(TMA) (DMSO-d6, 126 MHz)

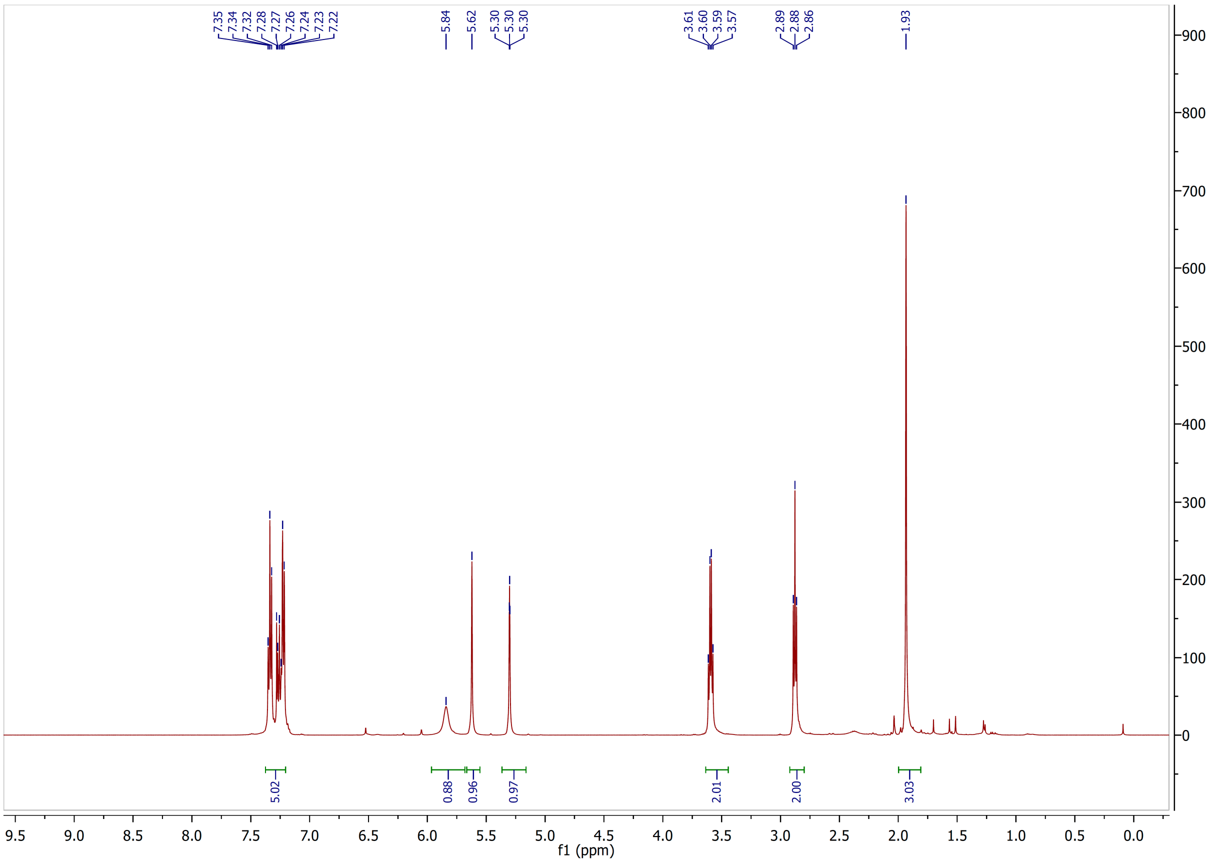

Figure S20: ^{1}H-NMR(PMA) ($CDCl_3$, 500 MHz)

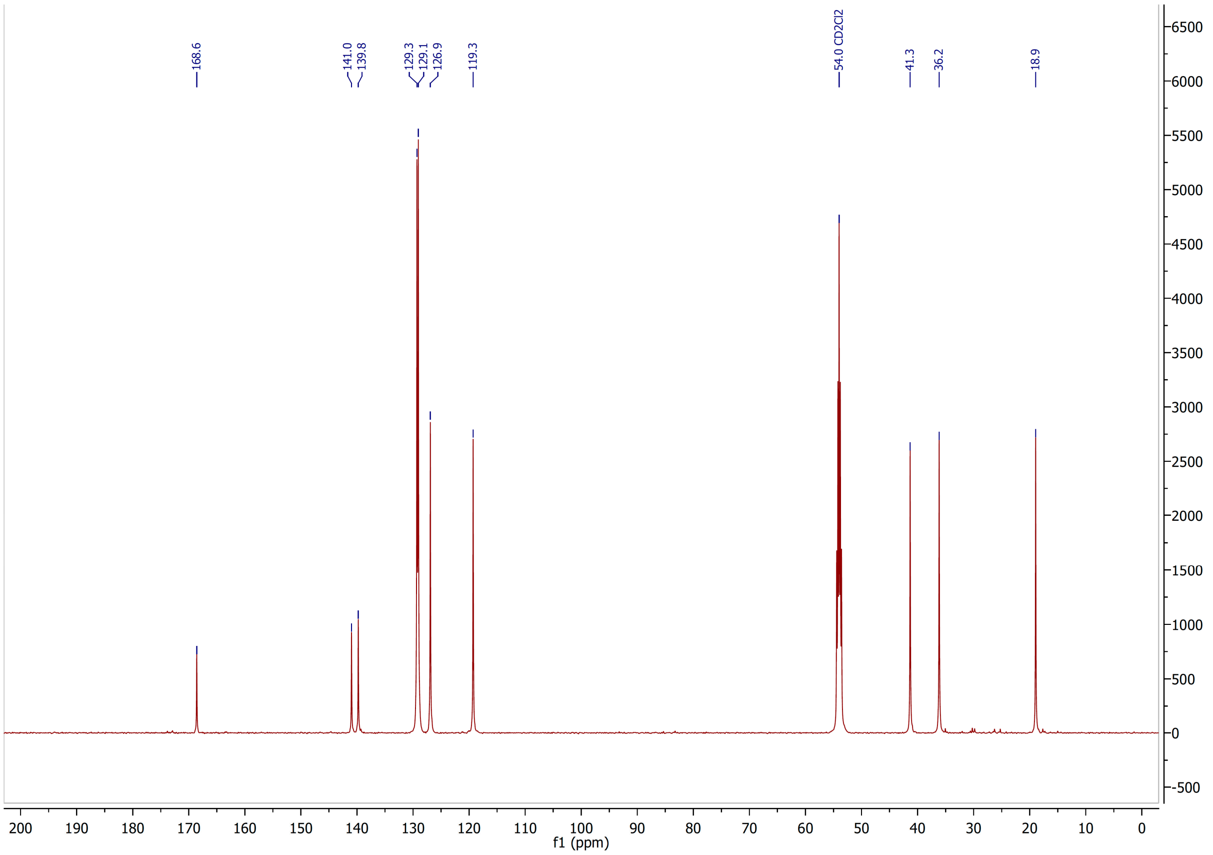

Figure S21: ^{13}C-NMR(PMA) (CD_2Cl_2, 126 MHz)

GPC:

GPC measurements from Waters GPC system with a column of polysulfone styrene. Chromatograms of the presented polymers are shown here, where the retention times where taken from and used for molecular weight calculations.

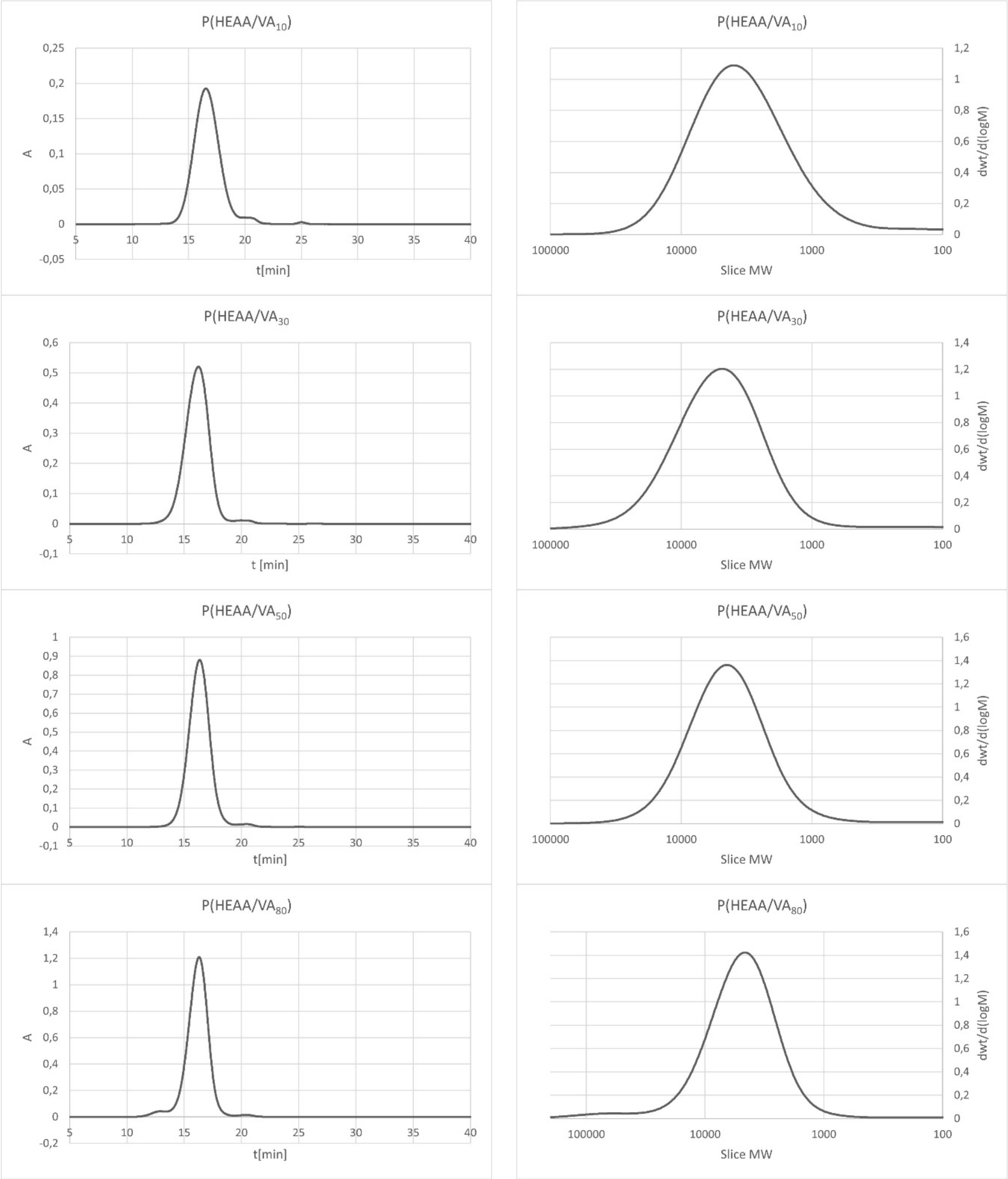

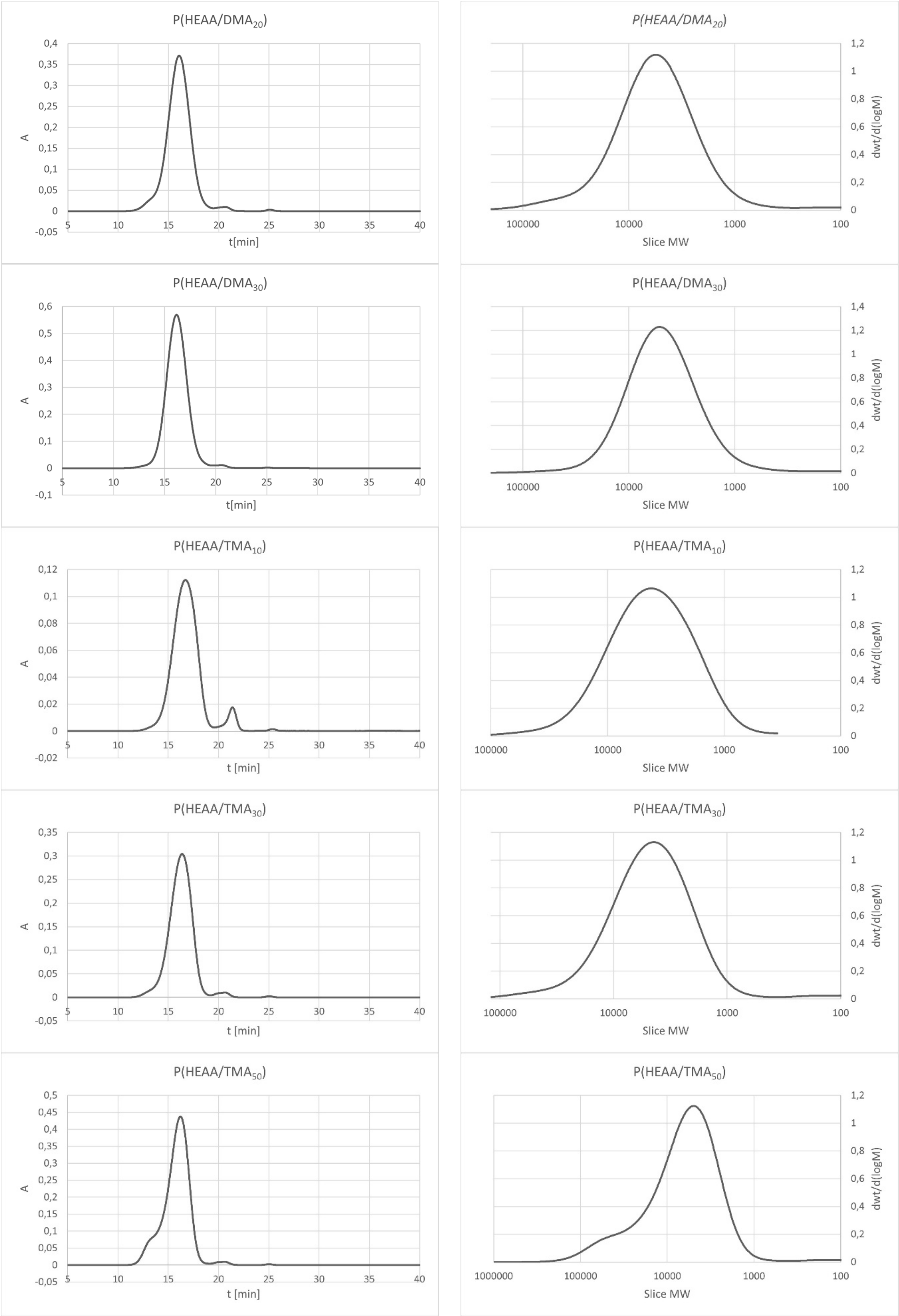

P(HEAA/DMA$_{20}$)
A
t[min]
P(HEAA/DMA$_{20}$)
dwt/d(logM)
Slice MW
P(HEAA/DMA$_{30}$)
A
t[min]
P(HEAA/DMA$_{30}$)
dwt/d(logM)
Slice MW
P(HEAA/TMA$_{10}$)
A
t [min]
P(HEAA/TMA$_{10}$)
dwt/d(logM)
Slice MW
P(HEAA/TMA$_{30}$)
A
t [min]
P(HEAA/TMA$_{30}$)
dwt/d(logM)
Slice MW
P(HEAA/TMA$_{50}$)
A
t [min]
P(HEAA/TMA$_{50}$)
dwt/d(logM)
Slice MW

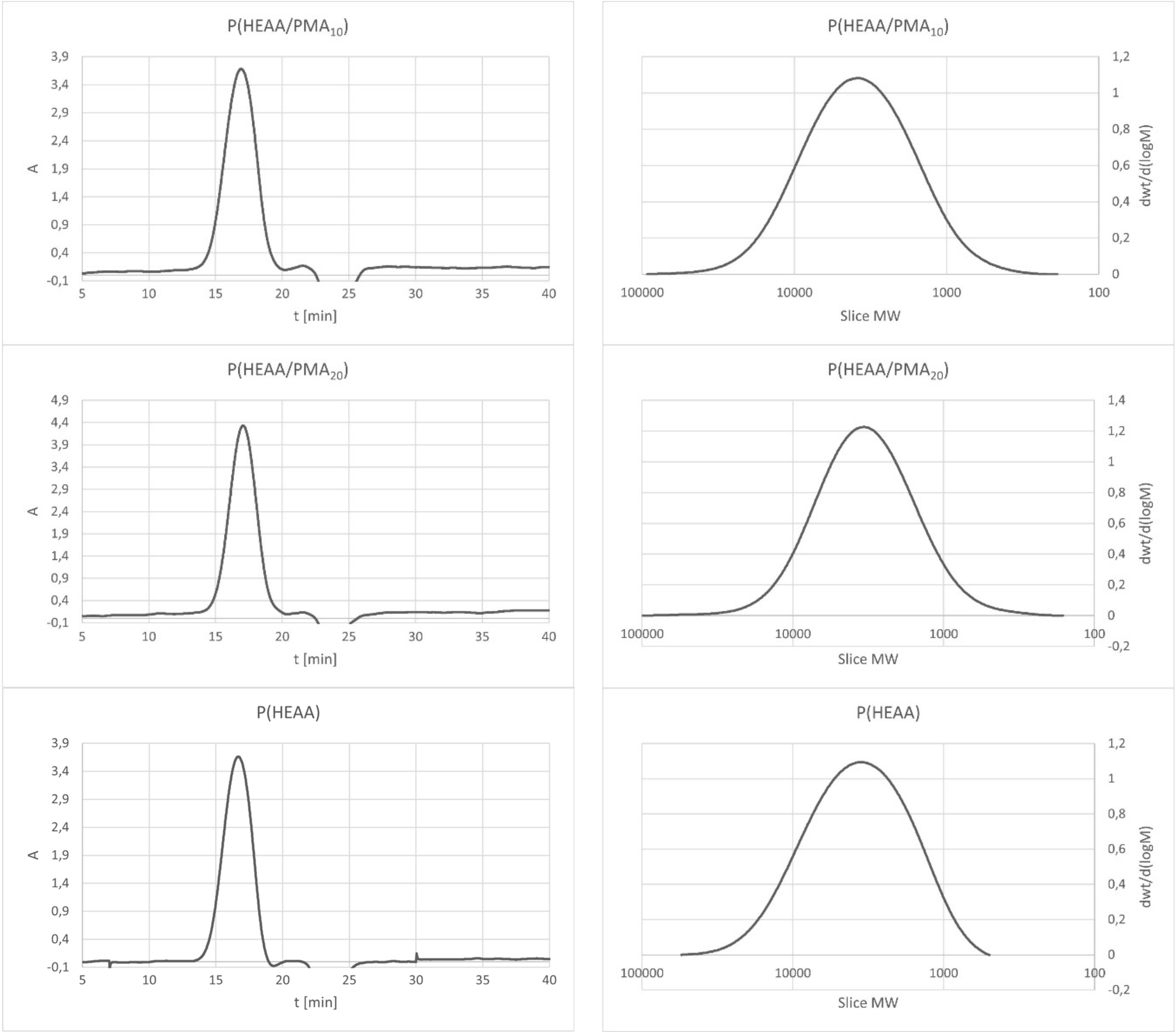

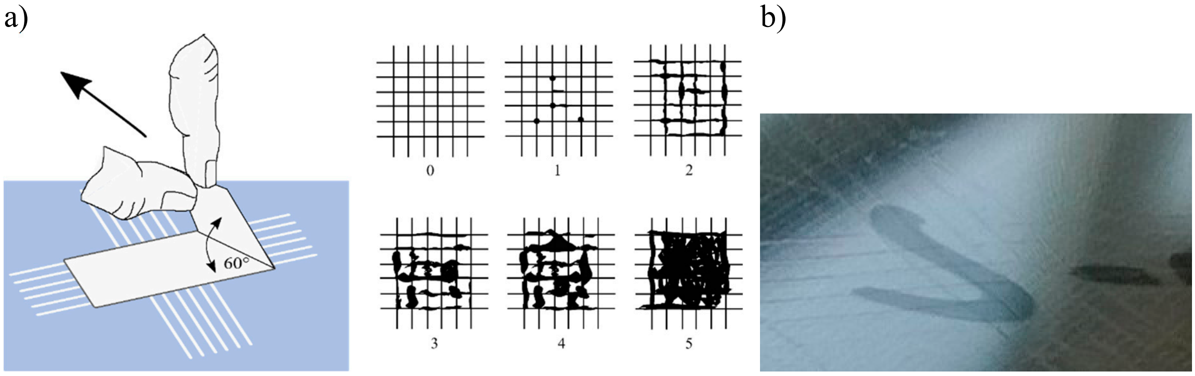

Adhesion test: a) Cross-cut test according to DIN EN ISO 2409: ISO scale 0 - 5, 0 = cut edges clean and no detachment of grid squares, 1 = less than 5% of test area affected, 2 = 5 - 15%; 3 = 15 - 35%, 4 = 35 - 65%, 5 = higher than 65% of test area affected, b) adhesion test: P(HEAA/DMA$_{10}$) on glass: cross-cut class 0. Only one tested sample was photo-documented.
